# Histological types of invasive breast cancer in 830,000 women diagnosed in England during 1988–2016

**DOI:** 10.1002/2056-4538.70043

**Published:** 2025-09-18

**Authors:** Jake Probert, John Broggio, Sarah C Darby, David Dodwell, Paul McGale, Carolyn Taylor, Kezia Gaitskell

**Affiliations:** ^1^ Nuffield Department of Population Health University of Oxford Oxford UK; ^2^ The National Disease Registration Service NHS England Leeds UK; ^3^ Department of Oncology Oxford University Hospitals NHS Foundation Trust Oxford UK; ^4^ Nuffield Division of Clinical Laboratory Sciences, Radcliffe Department of Medicine University of Oxford Oxford UK; ^5^ Department of Cellular Pathology Oxford University Hospitals NHS Foundation Trust Oxford

**Keywords:** breast cancer, incidence, histological type, molecular subtype

## Abstract

Breast cancer can be categorised into a number of histological types, based on microscopic appearances. There is some evidence that the different breast cancer histological types are associated with different patient and tumour characteristics, but few previous studies have been large enough to investigate this systematically, especially for rare histological types. National cancer registration data were used to describe trends in the incidence of specific histological types of invasive breast cancer in women diagnosed when aged 18–89 years in England from January 1988 to December 2016, and to investigate associations between breast cancer histological types and patient and tumour characteristics. There were 838,776 women diagnosed with a first primary invasive breast cancer in this 29‐year period, including 614,698 (73%) cases of ductal carcinoma NST [no special type (NST)], 90,028 (11%) cases of lobular carcinoma, and more than 16,000 (2%) cases each of tubular and mucinous carcinomas. Rarer histological types included medullary, papillary, metaplastic, and cribriform carcinomas, with >1000 cases of each type. Data quality and completeness improved substantially during the study period. The different histological types of breast cancer showed different patterns in incidence by calendar period of diagnosis, age at diagnosis, and screen‐detection status, as well as different associations with tumour characteristics such as grade, stage at diagnosis, and molecular subtype. This large nationwide study provides an overview of the changing incidence of the different histological types of invasive breast cancer in England over almost 30 years. It also gives an opportunity to investigate the characteristics of rare histological types, which smaller studies have been unable to explore. In addition, the results demonstrate the continuing value of histological types defined by microscopic morphology, alongside newer molecular classifications.

## Introduction

Breast cancer can be categorised into multiple histological types that were originally defined based on microscopic appearances (i.e., morphology) [1, 2, 3, 4, 5]. The most common is invasive ductal carcinoma of no special type (NST), which encompasses most primary breast cancers not showing features of any special type. Of the special types of invasive breast carcinoma, the most common is invasive lobular. Other special types include tubular, cribriform, micropapillary, papillary, metaplastic, mucinous, apocrine, adenoid cystic, and medullary [3, 6].

In the past two decades, this histological classification of breast cancer has been complemented by intrinsic molecular subtypes, defined by gene expression data [7, 8]. As such data are typically not available except in certain research settings, approaches have been developed to approximate the molecular subtypes using a small panel of immunohistochemical markers including oestrogen receptor (ER), progesterone receptor (PR), human epidermal growth factor receptor (HER2), and the proliferation marker Ki‐67 [9]. Information on immunohistochemical staining is typically cheaper and easier to obtain than gene expression data, and some immunohistochemical staining is routinely performed in clinical practice. Molecular classifications vary but typically include: ‘Luminal A’, ‘Luminal B (HER2 negative)’, ‘Luminal B (HER2 positive)’, ‘HER2 positive (non‐luminal)’, and ‘Triple negative’ or ‘basal‐like’ [9].

Previous studies have suggested that these various breast cancer histological types and molecular subtypes may result from distinct patterns of reproductive, lifestyle, and genetic risk factors [10, 11, 12, 13, 14, 15, 16, 17, 18], and may also have different prognoses [19, 20]. Some studies have also explored the changing incidence of the different breast cancer histological types and molecular subtypes over time [20, 21]. However, there have been few population‐based studies large enough to explore detailed tumour characteristics for the histological types, especially rarer subtypes [22].

We used national cancer registration data to describe trends in the incidence of specific histological types of invasive breast cancer in women diagnosed in England over a 29‐year period and to investigate associations between breast cancer histological types and other patient and tumour characteristics.

## Materials and methods

### Study population

Data were obtained from NHS England's Data Access Request Service on all 956,811 women registered with breast cancer as their first invasive cancer in England during January 1988 to December 2016. These data included date of diagnosis, age at diagnosis, screen‐detection status (i.e., whether or not the cancer was detected via the breast‐screening programme), tumour size, number of positive nodes, stage, grade, and hormone receptor status (ER, PR, and HER2). Further details on the population and methods are given elsewhere [23].

A total of 118,035 women were then excluded (supplementary material, Figure S1) because: they were aged <18 or 90+ years at diagnosis, their registrations were based on death certificates only, or they died or were lost to follow‐up within 3 months of diagnosis (as such patients would be unlikely to have a full diagnostic work‐up including surgery, and so would have incomplete information). Analyses were restricted to women with invasive primary breast cancer, that is, excluding *in situ*, encapsulated/encysted, or microinvasive carcinomas; haematological malignancies; primary skin tumours; or tumours metastatic from other sites. Malignant mesenchymal tumours/sarcomas were excluded but metaplastic carcinomas were included. Other exclusions were women who had a second primary cancer or contralateral cancer within 3 months of diagnosis (as it might be unclear which of the two tumours data related to). This left a total of 838,776 women in the study.

Data on histological type were reviewed by a pathologist (KG). Thirteen main histological groups were derived, including ductal carcinoma of NST and 11 ‘special types’ of carcinoma (lobular, mucinous, tubular, carcinoma with medullary features, papillary, metaplastic, cribriform, carcinoma with apocrine differentiation, micropapillary, adenoid cystic, and carcinoma with neuroendocrine features) [see supplementary material, Figure S2, Text S1, and Tables S1 and S2 for details of the histopathology classification based on the World Health Organization (WHO) classification of breast tumours [1, 2, 3] and codes from the International Classification of Diseases for Oncology (ICD‐O) [24, 25, 26]]. Despite its usual definition as a clinical pattern rather than a specific histological type [3], inflammatory carcinomas were also included as a separate histopathological group when coded as such in the cancer registry data. Histological types with fewer than 200 cases in the analysis population were grouped together to form a category ‘Other’. Unspecified malignant tumours were grouped separately. Tumours of mixed type which involve ductal carcinoma NST (e.g., ductal carcinoma and lobular carcinoma or other types) were grouped with ductal carcinoma NST. Tumours of mixed lobular carcinoma and other (non‐ductal) types were grouped with lobular carcinoma.

Cancers were categorised to reflect eligibility for the breast cancer screening programme based on age and calendar year; the eligible group was then categorised as ‘screen‐detected’, ‘interval‐detected’ or ‘other’. Intrinsic molecular subtype was estimated using hormone receptor status and grade. Based on St Gallen's surrogate definitions [9], grade was used in place of Ki‐67 (see supplementary material, Table S3).

The information received from NHS England was not accompanied by detailed data as to the source of the information. For example, it is unclear whether the histological type was based on information from the original diagnostic biopsy or from a subsequent cancer resection, and whether the tumour characteristic information (including tumour histological type, stage, and grade) came from a specimen prior to any therapy or following neoadjuvant chemotherapy.

### Method of analysis

To convert the numbers of women with breast cancer into incidence rates, mid‐year estimates for the female population of England by calendar year and single years of age were obtained from the Office of National Statistics [27]. Age‐specific incidence rates and 95% confidence intervals were calculated for each histological type, assuming the observed cases had a Poisson distribution. Tests for a trend with age in the incidence rates for each histological type used the likelihood ratio. Incidence rate ratios were estimated with group‐specific 95% confidence intervals [28].

Age‐standardisation was carried out by the direct method [29] using the 2013 European standard population. Age‐standardised incidence rates, rate ratios and 95% confidence intervals were calculated by histological type and calendar period of diagnosis, using the ‘distrate’ [30] command in Stata [31]. Trends in incidence rates by calendar period of diagnosis were presented graphically using a 3‐year moving average (i.e., the average of three consecutive years used to estimate the middle year).

Data completeness in tumour characteristics (especially hormone receptor status) was poor for women diagnosed in the early calendar periods. Therefore, when examining trends in such characteristics, to reduce the effect of missing data and to avoid large changes over calendar periods due just to increasing data completeness, some analyses focussed on women diagnosed more recently. Hence, the percentage distributions of tumour characteristics were examined in women diagnosed during 2010–2016. Where data were still missing, separate categories were created for the missing values. All calculations were performed using Stata version 18.1 [31]; figures were plotted in R [32].

### Ethics approval statement

The study was approved by NHS England's Data Access Request Service (reference DARS‐NIC‐656816‐Z3N6R‐v1.4).

### Patient consent statement

The study uses routinely collected data that was de‐personalised before release. Informed consent from individual participants was not required.

## Results

### Characteristics of study population

Among the 838,776 eligible women diagnosed with invasive breast cancer during 1988–2016, 73.3% (614,698) had ductal carcinoma NST. Lobular carcinoma accounted for 10.7% of cases (90,028), mucinous carcinoma for 2.0% (16,361), tubular carcinoma for 1.9% (16,118), and medullary carcinoma for 0.5% (4295) (Table 1). Rare histological types, each accounting for <0.5% of cases, were papillary (3295), metaplastic (2250), cribriform (1617), apocrine (951), micropapillary (763), adenoid cystic (509), neuroendocrine (463), inflammatory (354), and other (414). During 1988–2009, 13.6% of breast cancers were recorded as ‘unspecified’ histological type, but only 2.5% in 2010–2016.

**Table 1:** Characteristics of 838,776 women diagnosed with invasive breast cancer in England during 1988–2016, by diagnosis period

Characteristic	Number (%) of women by calendar period of breast cancer diagnosis		Total
	1988–2009	2010–2016	
Histological type (*p* < 0.001)*			
Ductal^†^	416,243 (70.3)	198,455 (80.5)	614,698 (73.3)
Lobular	61,627 (10.4)	28,401 (11.5)	90,028 (10.7)
Unspecified	80,342 (13.6)	6318 (2.5)	86,660 (10.3)
Mucinous	11,401 (1.9)	4960 (2.0)	16,361 (2.0)
Tubular	12,125 (2.0)	3993 (1.6)	16,118 (1.9)
Medullary	3868 (0.7)	427 (0.2)	4295 (0.5)
Papillary	2371 (0.4)	924 (0.4)	3295 (0.4)
Metaplastic	1225 (0.2)	1025 (0.4)	2250 (0.3)
Cribriform	1346 (0.2)	271 (0.1)	1617 (0.2)
Apocrine	465 (0.1)	486 (0.2)	951 (0.1)
Micropapillary	78 (<0.1)	685 (0.3)	763 (0.1)
Adenoid cystic	343 (0.1)	166 (0.1)	509 (0.1)
Neuroendocrine	299 (0.1)	164 (0.1)	463 (0.1)
Other	274 (<0.1)	140 (0.1)	414 (<0.1)
Inflammatory	292 (<0.1)	62 (<0.1)	354 (<0.1)
Age at diagnosis, years (*p* < 0.001)			
18–39	34,936 (5.9)	12,081 (4.9)	47,017 (5.6)
40–49	98,204 (16.6)	42,516 (17.2)	140,720 (16.8)
50–64	224,946 (38.0)	89,456 (36.3)	314,402 (37.5)
65–70	75,655 (12.8)	39,228 (15.9)	114,883 (13.7)
71–79	94,667 (16.0)	35,686 (14.5)	130,353 (15.5)
80–89	63,891 (10.7)	27,510 (11.2)	91,401 (10.9)
Cancer screen detected^‡^ (*p* < 0.001)			
Eligible: screen detected	88,568 (15.0)	66,423 (26.9)	154,991 (18.5)
Eligible: not screen detected (interval cancer)	34,520 (5.8)	28,640 (11.6)	63,160 (7.5)
Eligible: not screen detected (other)	124,745 (21.1)	33,621 (13.6)	158,366 (18.9)
Not eligible for screening	344,466 (58.1)	117,793 (47.8)	462,259 (55.1)
Tumour grade (*p* < 0.001)			
Low	79,657 (13.4)	36,491 (14.8)	116,148 (13.8)
Medium	194,359 (32.8)	122,510 (49.7)	316,869 (37.8)
High	149,387 (25.2)	78,237 (31.7)	227,624 (27.1)
Unknown	168,896 (28.6)	9239 (3.8)	178,135 (21.3)
Stage (*p* < 0.001)			
Local	91,283 (15.4)	90,552 (36.7)	181,835 (21.7)
Regional	102,254 (17.3)	101,842 (41.3)	204,096 (24.3)
Metastatic	8666 (1.5)	9368 (3.8)	18,034 (2.2)
Unknown	390,096 (65.8)	44,715 (18.2)	434,811 (51.8)
Tumour size (*p* < 0.001)			
1–20 mm	191,415 (32.3)	110,451 (44.8)	301,866 (36.0)
21–50 mm	127,389 (21.5)	74,193 (30.1)	201,582 (24.0)
>50 mm	17,244 (2.9)	10,590 (4.3)	27,834 (3.3)
Unknown	256,251 (43.3)	51,243 (20.8)	307,494 (36.7)
Number of positive nodes (*p* < 0.001)			
0	94,964 (16.0)	113,323 (46.0)	208,287 (24.8)
1–3	59,684 (10.1)	46,028 (18.7)	105,712 (12.6)
4–9	21,437 (3.6)	11,305 (4.6)	32,742 (3.9)
10 or more	9580 (1.6)	5691 (2.3)	15,271 (1.8)
Unknown	406,634 (68.7)	70,130 (28.4)	476,764 (56.9)
ER status (*p* < 0.001)			
Negative	8871 (1.5)	26,754 (10.9)	35,625 (4.2)
Positive	42,625 (7.2)	152,342 (61.8)	194,967 (23.2)
Unknown	540,803 (91.3)	67,381 (27.3)	608,184 (72.6)
PR status (*p* < 0.001)			
Negative	7556 (1.3)	30,140 (12.2)	37,696 (4.5)
Positive	14,208 (2.4)	62,365 (25.3)	76,573 (9.1)
Unknown	570,535 (96.3)	153,972 (62.5)	724,507 (86.4)
HER2 status (*p* < 0.001)			
Negative	20,014 (3.4)	155,930 (63.3)	175,944 (21.0)
Positive	6874 (1.2)	29,663 (12.0)	36,537 (4.4)
Unknown	565,411 (95.4)	60,884 (24.7)	626,295 (74.6)
Total	592,299 (100)	246,477 (100)	838,776 (100)

Information on region, deprivation index, and ethnicity is given in supplementary material, Tables S6, S7, S10, and S11.

* The *p* values are for *χ* ^2^ tests of independence between characteristic and calendar period of diagnosis.

^†^ Ductal carcinoma of no special type.

^‡^ Categories reflect eligibility for breast cancer screening programme (i.e., 50–64 years for women diagnosed in 1988–2004 and 50–70 years for 2005–2016). Interval detected cancers are those diagnosed during the 3 years after a normal screening result and before the next screening appointment.

Women aged 50–70 at diagnosis (the age‐range at which breast cancer screening is offered routinely in the UK at present) accounted for 51.2% of cases, whilst women younger than 50 and older than 70 made up 22.4% and 26.4%, respectively. During 1988–2009, 15.0% of cancers were detected via the routine breast screening programme, increasing to 26.9% in 2010–2016. The proportion of interval breast cancers also increased, from 5.8% in 1988–2009 to 11.6% in 2010–2016. These changes are due largely to the expansion of screening to include women aged between 65 and 70, which began in some screening centres in 2001 and was implemented nationwide in England from 2005 [33].

### Histological types – incidence by age

**Figure 1:**
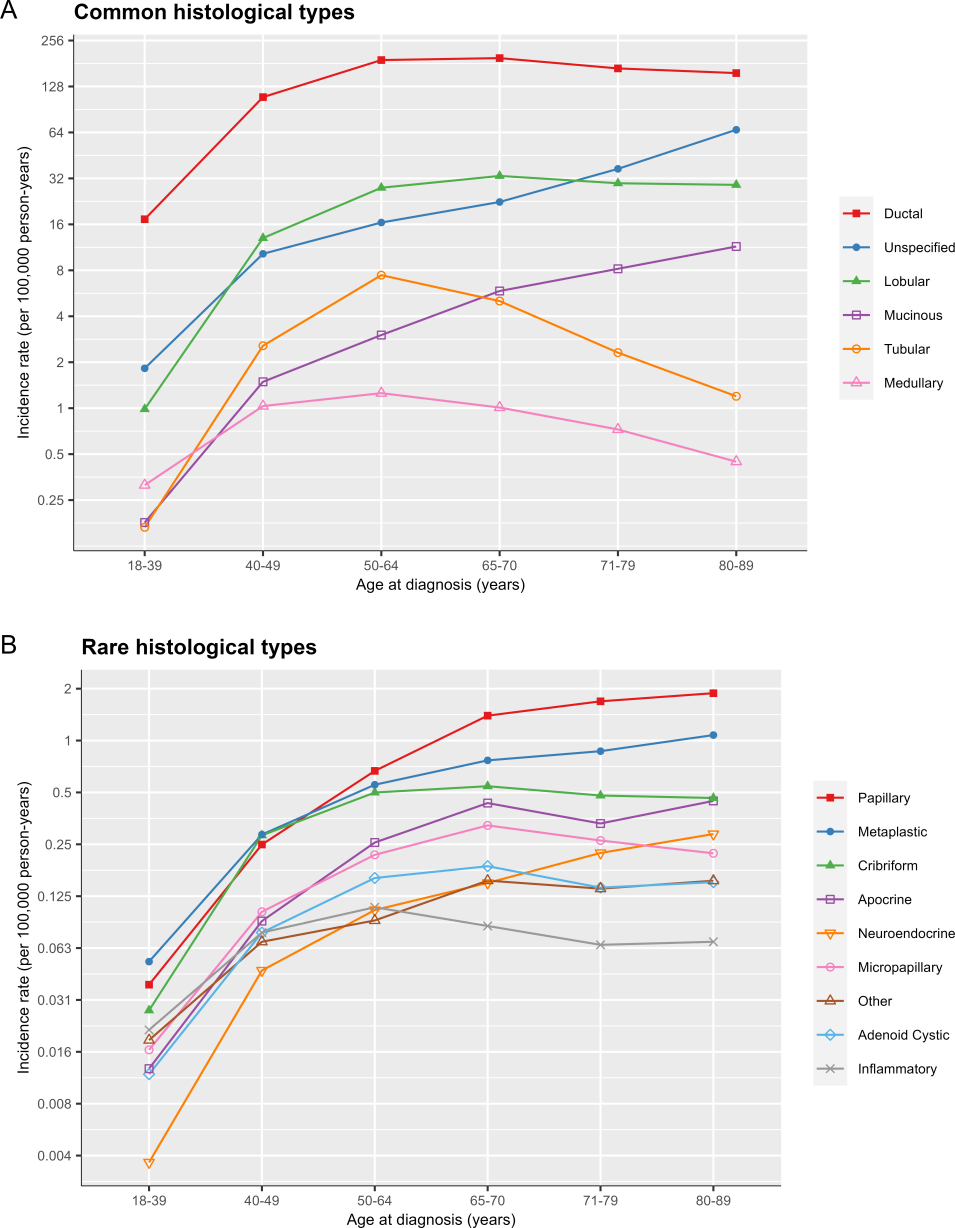
Age‐specific incidence rates of invasive breast cancer types in 838,776 women diagnosed during 1988–2016. The incidence rates on the y‐axis are plotted on a log scale and the scales differ between panels (A) common histological types, and (B) rare histological types. Legends are ordered by incidence rates in women aged 80–89 years. See supplementary material, Table S4 for numbers.

Age‐specific incidence rates differed substantially between the histological types (Figure 1). As expected, for most types, the incidence rate increased with increasing age and often plateaued after about age 70, coinciding with the end of routine screening. Above age 70, the incidence of tubular and medullary carcinomas fell, but the incidence of mucinous and neuroendocrine carcinomas continued to rise, as did the incidence of cases registered as ‘Unspecified’ histological type. In women aged ≤70, fewer than 10% of breast cancers were of ‘Unspecified’ type, rising to 14.7% at age 71–79 and 24.7% at age 80–89 (supplementary material, Tables S4–S7).

### Histological types – association with screening

**Figure 2:**
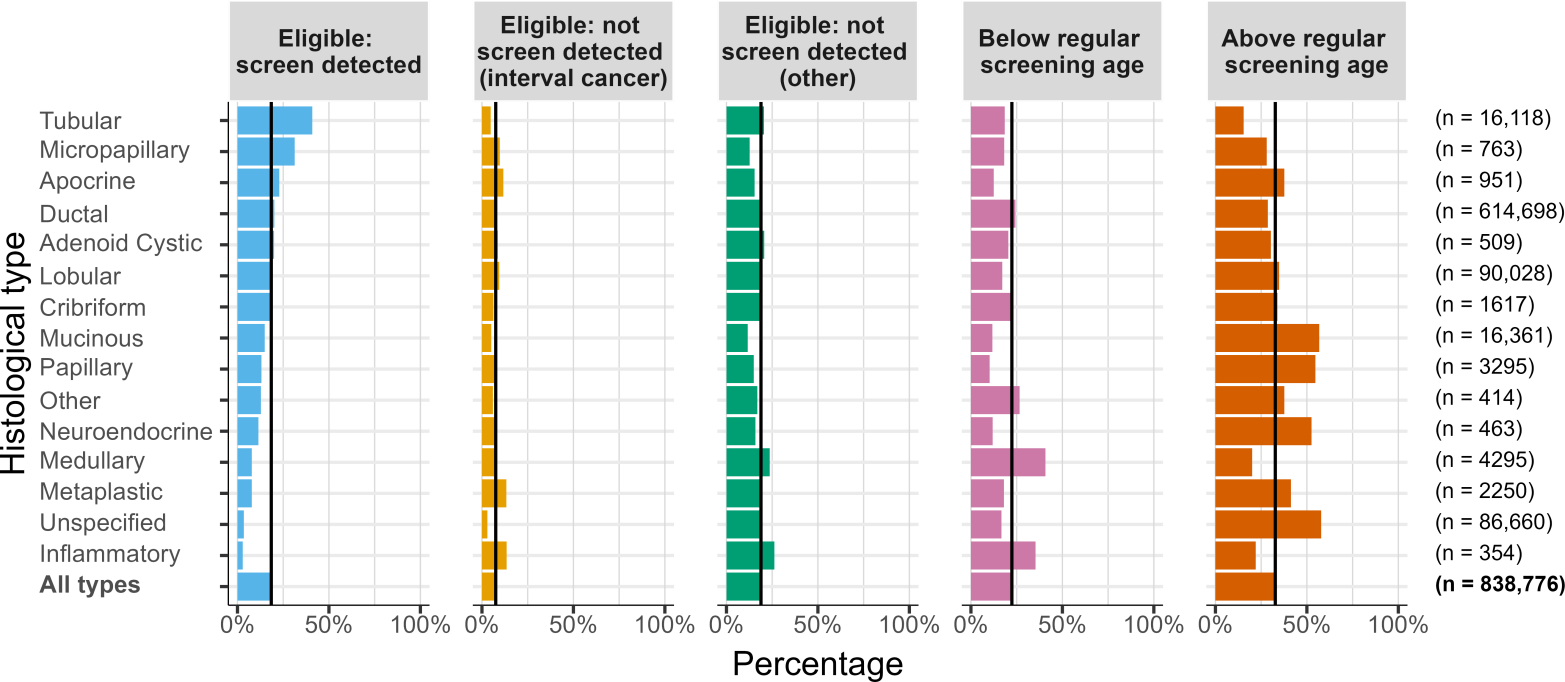
Percentage of cases detected by screening within each histological type of invasive breast cancer for 838,776 women diagnosed during 1988–2016. The eligibility for the breast cancer screening programme was 50–64 years for women diagnosed in 1988–2004 and 50–70 years for 2005–2016. Histological types are ordered by the percentage of screen‐detected cancers. The vertical lines show percentages for all types combined. See supplementary material, Table S8 for numbers.

To further assess associations between breast screening and diagnosis of specific breast cancer types, we explored the proportion of cases of each type diagnosed through screening according to whether or not the women were in age groups eligible for screening. There were substantial variations in the distribution of breast cancer histological types by screen detection status (Figure 2 and supplementary material, Table S8). For example, 40.9% of tubular carcinomas were diagnosed through screening, compared to 20.2% of ductal carcinomas, 7.8% of medullary and metaplastic carcinomas, and 3.4% of cancers of ‘unspecified’ type.

### Histological types – by calendar period

**Figure 3:**
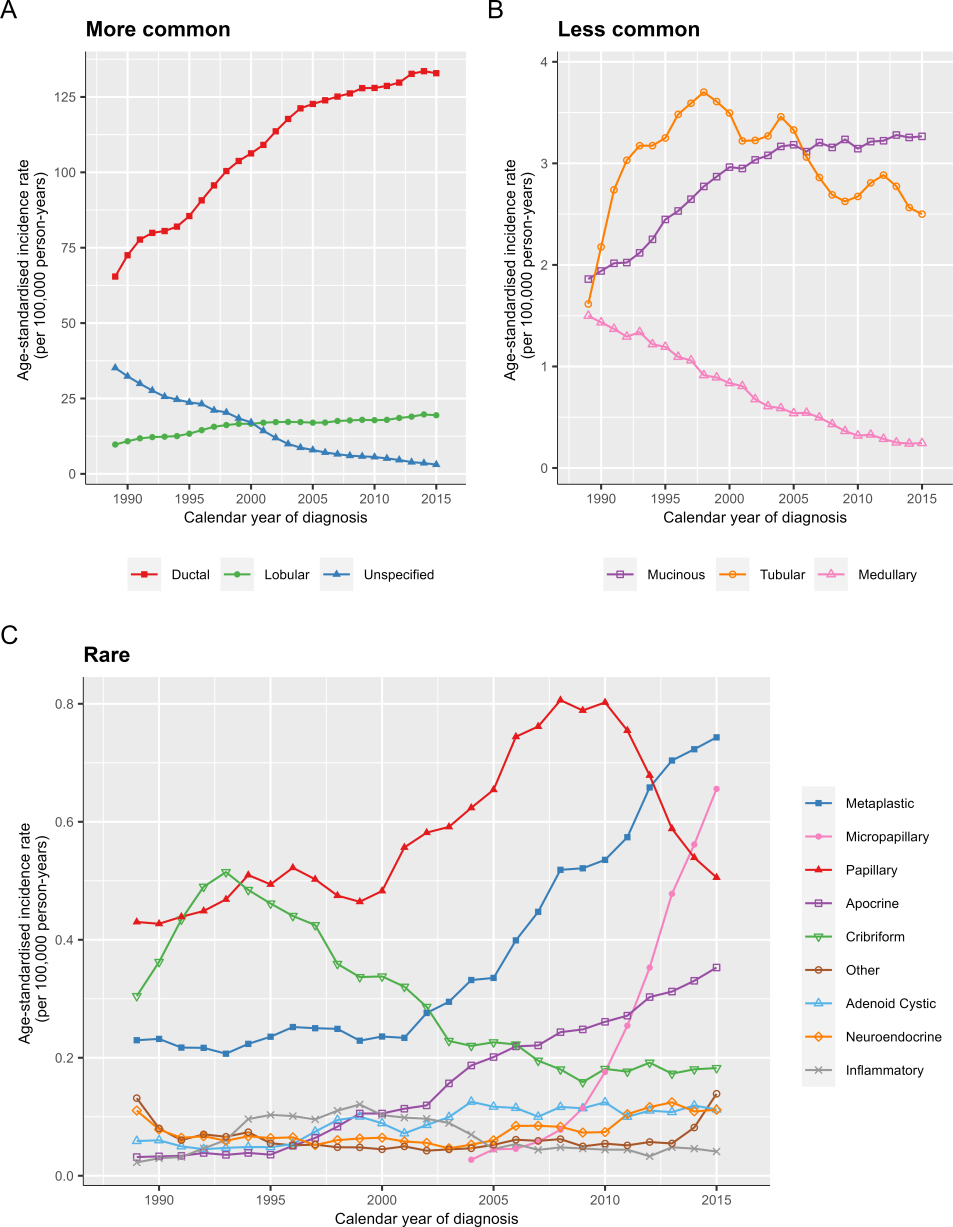
Age‐standardised incidence rates of invasive breast cancer types by calendar year of diagnosis in 838,776 women diagnosed during 1988–2016. Incidence rates are standardised to 2013 European standard population and smoothed using a 3‐year moving average. The scales on the y‐axis differ between panels (A) more common types, (B) less common types, and (C) rare types. Legends are ordered by standardised incidence rate for women diagnosed in the most recent period. See supplementary material, Tables S9i–S9xv for numbers.

To assess changes in the incidence of breast cancer histological types over time, we calculated age‐standardised incidence rates for different types by calendar period. Among the more common types, the incidence of ductal carcinoma approximately doubled during the study period, rising from 57.5 per 100,000 person‐years in 1988 to 131.0 in 2016 (Figure 3 and supplementary material, Table S9). The incidence of lobular carcinoma also approximately doubled, from 8.4 per 100,000 person‐years in 1988 to 18.7 in 2016.

Among less‐common types, the incidence of medullary carcinoma reduced by a factor of five over the study period, from 1.4 per 100,000 person‐years in 1988 to 0.27 in 2016. By contrast, the incidence of mucinous carcinomas increased from 1.9 per 100,000 person‐years in 1988 to 3.2 in 2016. The incidence of tubular carcinomas increased during the 1990s, from 1.0 per 100,000 person‐years in 1988 to 3.8 in 1997, but has fallen somewhat since then.

Among rare types, micropapillary carcinomas have been reported only since 2003, but the incidence has increased substantially since then, rising from 0.01 per 100,000 person‐years in 2003 to 0.72 in 2016. By contrast, the incidence of papillary carcinomas rose in the early 2000s, but then fell after the 2009–2011 period, coinciding with the increase in micropapillary carcinomas. The reported incidence of cribriform carcinomas fell in the late 1990s and stabilised post 2003. Other rare types for which incidence patterns changed over time include metaplastic carcinoma (incidence tripled since 2000) and apocrine carcinoma (incidence increased 15‐fold).

### Histological types – variation in tumour characteristics

**Figure 4:**
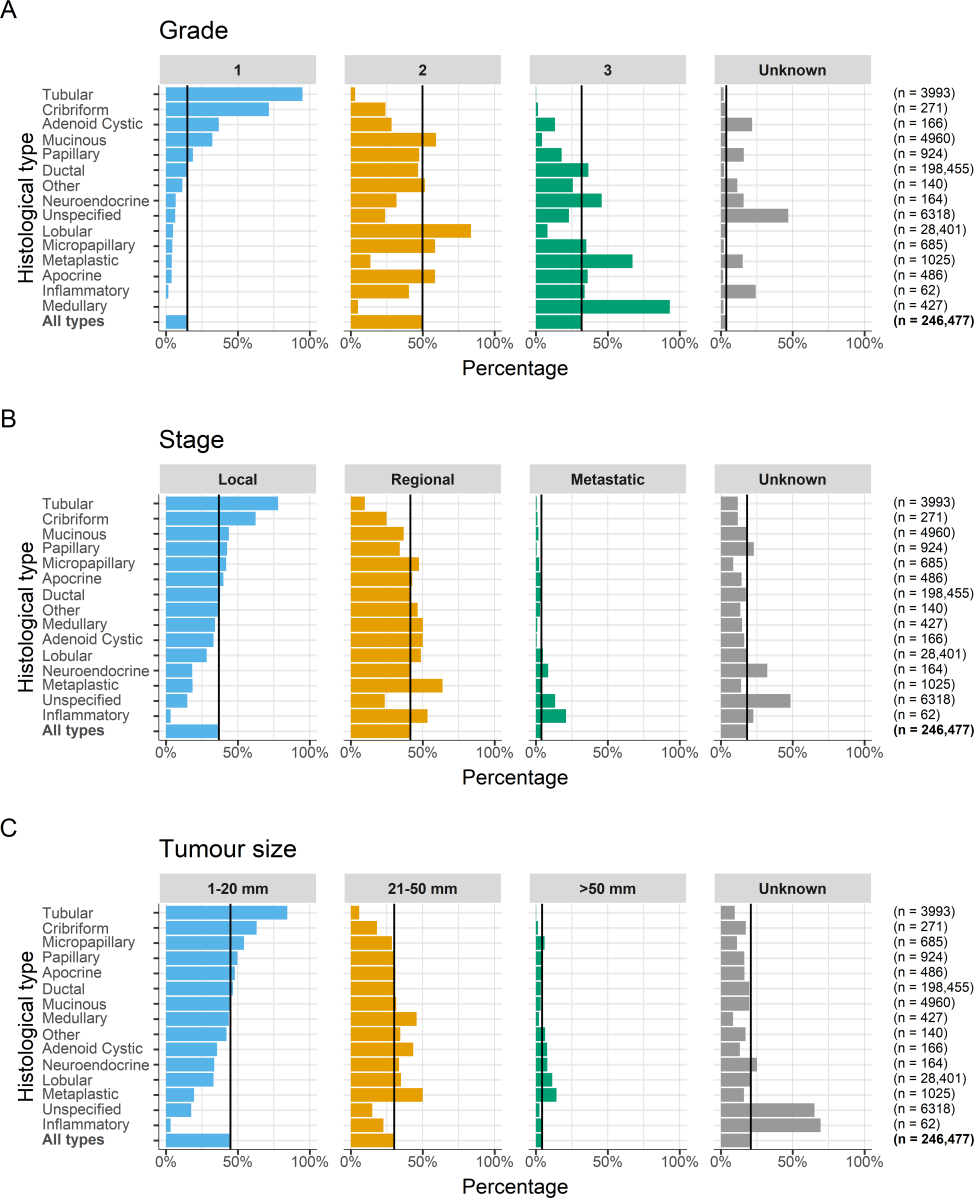
Percentage of (A) grade, (B) stage, and (C) tumour size within each histological type of invasive breast cancer for 246,477 women diagnosed during 2010–2016. Histological types are ordered by the percentage of (A) grade 1 tumours, (B) local tumours, and (C) 1–20 mm tumours. The vertical lines show percentages for all types combined. See supplementary material, Tables S10 and S11 for numbers.

We assessed the variation in tumour characteristics between different histological types of invasive breast cancer during 2010–2016 (Figure 4 and supplementary material, Tables S10 and S11). During this more recent period, the information on tumour characteristics (namely grade, stage, tumour size, and number of positive nodes) was much more complete, with the percentage of unknowns ranging from 4% to 28%, compared to 29% to 69% during 1988–2009 (Table 1). Despite this improvement, information on tumour characteristics was still unknown for between 47% and 72% in those with ‘Unspecified’ histological type in the 2010–2016 period.

For tumour grade, there was substantial variation by histological type, as expected. For example, 95.0% of tubular and 71.6% of cribriform carcinomas were Grade 1, whereas 93.2% of medullary and 67.2% of metaplastic carcinomas were Grade 3. For ductal carcinoma, 14.5% were Grade 1, 46.7% Grade 2, and 36.5% Grade 3. For lobular carcinoma, 4.9% were Grade 1, 83.6% Grade 2, and 8.1% Grade 3.

For stage at diagnosis and tumour size, there was also substantial variation by tumour histological type. For example, local disease stage at diagnosis was common for cases of tubular carcinoma (78.0%) and cribriform carcinoma (62.4%), whereas regional spread was more common for cases of metaplastic carcinoma (63.7%), and 21.0% of cases of inflammatory breast cancer presented with metastatic disease. Similarly, a tumour size of ≤20 mm was relatively common in tubular carcinomas (84.4%) and cribriform carcinomas (63.1%) but was uncommon in metaplastic (19.6%) or inflammatory (3.2%) carcinomas.

**Figure 5:**
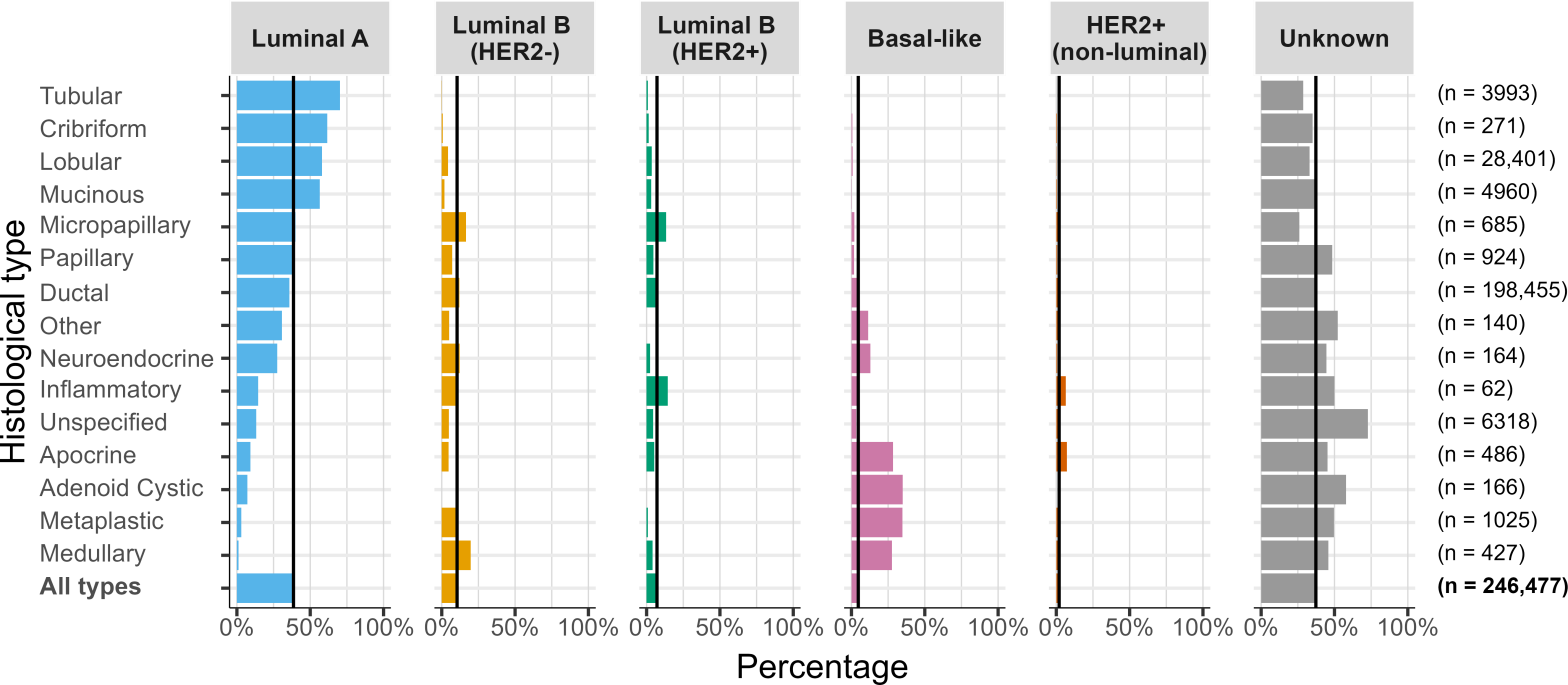
Percentage of molecular subtype within each histological type of invasive breast cancer for 246,477 women diagnosed during 2010–2016. Histological types are ordered by the percentage of Luminal A cancers. The vertical lines show percentages for all types combined. See supplementary material, Table S3 for definitions and Table S12 for numbers.

For molecular subtype, we assessed distribution across different histological types (Figure 5 and supplementary material, Table S12). Even within the period 2010–2016, hormone receptor status was often missing from these national data: ER status was unknown in 27.3%, PR status in 62.5%, and HER2 status in 24.7% (supplementary material, Table S10). Where information enabling molecular subtype classification was available, the distribution of molecular subtypes varied by histological type. The luminal A subtype was common in cases of tubular, cribriform, lobular, and mucinous carcinomas, while the basal‐like (triple‐negative) subtype was more common in apocrine, adenoid cystic, metaplastic, and medullary carcinomas. Repeating the analysis by age group at diagnosis (<50, 50–70, 71+) or screen‐detection status yielded mostly similar results (supplementary material, Tables S13 and S14 and Figures S3 and S4).

Invasive ductal carcinoma, the commonest histological type, showed heterogeneous molecular subtypes: 35.9% were of Luminal A, 11.9% of Luminal B (HER2−), 8.0% of Luminal B (HER2+), 5.2% of Basal‐like, 2.3% HER2+ (non‐luminal), and 36.7% of unknown molecular type (see Figure 5 and supplementary material, Table S12). Within cases of invasive ductal carcinoma, molecular subtype varied by tumour characteristics (see supplementary material, Table S15).

## Discussion

This study provides an accurate picture of breast cancer incidence according to histological type in a complete population for over a quarter of a century. Comprising all 838,776 women registered with invasive breast cancer in England during 1988–2016, the study documents the patterns in breast cancer incidence with age and calendar period in 15 distinct histological types, and highlights the improvements in completeness of the data available within NHS England over that period. We also explore grade, stage, tumour size, and molecular subtype for each histological type for women diagnosed during 2010–2016. We show that different histological types have different patterns of characteristics. For example, tubular carcinomas tend to be small, low‐grade tumours, of luminal‐A molecular subtype, and are usually detected by screening. By contrast, metaplastic carcinomas tend to be larger, high‐grade tumours, of basal‐like molecular subtype, and are not usually diagnosed through screening.

In this study, we describe a rising incidence of breast cancer diagnoses overall, but have not investigated possible underlying causes. Many other epidemiological studies have identified non‐genetic risk factors for breast cancer [34], including higher body adiposity in postmenopausal women [35, 36], oral contraceptive pill use in premenopausal women [37], menopausal hormone therapy use [38], and alcohol intake [39]; breastfeeding is associated with a lower risk [40]. Overall, approximately 23% of breast cancer cases in England may be attributable to potentially modifiable risk factors [41]. Other reported reproductive risk factors, including earlier age at menarche, later age at menopause, lower parity, and later age at first birth [13], are less easily modifiable. It seems plausible that rising overall population exposure to some of these risk factors in recent decades (such as increasing prevalence of obesity [42]) may explain at least part of the observed increase in breast cancer incidence in England, but this is beyond the scope of the current study.

### Strengths and limitations

The key strength of this study is the whole‐population coverage for incident breast cancer over nearly three decades, enabling description of trends over time in the incidence of the different histological types and exploration of characteristics of rare special types of breast cancer in a way not possible in previous studies.

One limitation of our study was incomplete data on tumour characteristics, especially for cases diagnosed in earlier years and in older patients. From 1988 to 2016, there was a 10‐fold reduction in the reporting of ‘Unspecified’ histological type, probably due to more consistent reporting of histological type by pathologists and better data collection by the cancer registries. There was also an association with age, with 26.1% of cases at age 80–89 recorded as ‘unspecified’ histological type. This may be because older patients are less likely to have aggressive investigation and/or treatment, resulting in less detailed tumour information being available (e.g., some patients may undergo a diagnostic biopsy but not a surgical excision).

Incomplete information was a particular issue for hormone receptor status. Before 2005, data on HER2 receptor status was not routinely collected, and not until 2010 did the percentage missing drop below 50%. Information on PR status is even less complete, and even for cases diagnosed after 2010, the estimated intrinsic molecular subtype was available for less than 50% of the cohort. Also, information on menopausal status was unavailable, so any associations between specific histological types (e.g., lobular carcinoma) and oestrogen exposure could not be considered. However, menopausal status is strongly associated with age, which we did explore.

Another limitation is that some women may have received neoadjuvant chemotherapy (i.e., chemotherapy prior to surgery). A previous study in this population found that overall 7.6% of women with breast cancer were recorded as receiving neoadjuvant chemotherapy [23]. In such women, it is uncertain whether the routinely collected tumour characteristic information relates to the tumour before or after chemotherapy. This may affect the data, as chemotherapy can affect multiple tumour characteristics, including tumour grade, size, stage, hormone receptor status, and occasionally histological type [43, 44, 45]. Finally, another limitation is that this study was restricted to cases of breast cancer in women. Breast cancer is much less common in men than in women, but does occur and has been explored in detail by other studies [46, 47, 48].

### Context

During the time period of this study, there were three editions of the evolving official classification system for breast cancer from the WHO, issued in 1981 [1], 2003 [2], and 2012 [3]. The changes in guidelines between these editions are summarised in supplementary material, Table S1; based on this, we can note where some of the changing incidence trends align with the publication year.

The 2003 edition of the WHO breast cancer classification introduced cribriform carcinoma, micropapillary carcinoma, and neuroendocrine tumours as new entities. Hence, it is unsurprising that diagnoses of micropapillary carcinomas increase from this year. The coincident fall in diagnoses of papillary carcinoma could indicate that tumours previously labelled papillary carcinomas began to be diagnosed as micropapillary carcinomas. Interestingly, cribriform carcinomas were reported throughout the study period, despite only appearing in the WHO classification in 2003.

It is unclear whether the substantial increases in the reported incidences of metaplastic and apocrine carcinomas over the study period, and the apparent decline in medullary carcinomas, represent genuine changes in incidence, or whether these observations are artefacts of improved awareness among pathologists or of changing diagnostic criteria.

It is challenging, if not impossible in some cases, to tease apart ‘genuine’ associations and trends from artificial changes due to evolving classification systems and diagnostic fashions. These considerations will continue to be important in future analyses as a new edition of the breast cancer classification was published in 2019, which contains further changes. For example, medullary carcinomas are no longer considered to be a separate entity, but rather a variant of invasive breast carcinoma of NST with prominent tumour‐infiltrating lymphocytes [49]. Molecular characteristics are also increasingly used to define certain histological types – for example, the diagnostic criteria for cribriform carcinomas now include ER‐positivity and HER2‐negativity in addition to the characteristic morphological appearances [49].

The data are also likely to be affected by routine breast screening. For example, tubular carcinomas were common among screen‐detected cancers, while metaplastic carcinomas were rare among screen‐detected cancers. This may suggest that tubular carcinomas are more amenable to being diagnosed by screening, and metaplastic carcinomas less so – perhaps due to relatively slow or rapid disease courses, respectively. The reduction in the incidence of tubular carcinomas diagnosed in women beyond the age for routine screening may reflect either a genuine reduction in the incidence of these tumours at older ages, or it may be due to the indolent clinical course of these tumours, making existing tumours more likely to have been diagnosed already during previous screening, and making newly developing tumours less likely to be detected symptomatically (i.e., non‐screen‐detected) in older women. An alternative explanation might be that some tumours that start as small, low‐grade, tubular carcinomas have the potential to progress to larger, higher‐grade carcinomas of non‐tubular histological type if not detected early via screening and treated.

### Comparison with other studies

Although previous studies have attempted to look at patterns of tumour characteristics by histological type, few have been large enough to describe reliably the characteristics of rare special types of breast cancer. The USA SEER cancer registry provides one of the few datasets with comparable population size to explore these questions; however, previous analyses of these data have still been too small to explore rare histological types. For example, one analysis of clinicopathological characteristics of breast cancer histological types in the SEER dataset included approximately 243,000 cases of breast cancer, that is, less than a third of the number of cases in this study [22].

Another study based on over 270,000 patients with breast cancer in the SEER dataset explored the age distribution of different breast cancer histological types, including relatively rare tumour types, but did not look at trends by calendar period or other detailed tumour characteristics [50]. As the USA does not have a standardised national breast‐screening programme in the same way as the UK, the age distribution of histological types in the SEER study did not have the same pronounced changes in incidence of tumours associated with the ages of breast screening seen in our study, but was otherwise broadly similar.

Other population‐based studies which have investigated time trends in breast cancer incidence have often focused on molecular subtypes rather than on morphological histological types [51], or else have been limited to common histological types such as ductal and lobular carcinoma [1, 52], or have not investigated histological type [53, 54].

Arguably, the recent tendency in some studies to focus primarily on the molecular subtypes of breast cancer, without also taking account of the morphology (i.e., the microscopic appearance), ignores information that is clinically and prognostically relevant. For example, we have shown that the ‘basal‐like’ (triple negative) molecular subtype is the most common for apocrine, adenoid cystic, metaplastic, and medullary carcinomas, and yet these histological types have quite different patterns of tumour characteristics (e.g., almost half of apocrine carcinomas are diagnosed with a tumour size ≤20 mm, compared to only a fifth of metaplastic carcinomas). While we did not address outcomes directly in this paper, factors such as smaller tumour size and lower grade are typically strongly associated with better prognosis [23].

### Future research

It is perhaps unsurprising that tumour morphology captures clinically important information in addition to molecular subtyping, as the tumour microscopic appearances are arguably an end‐phenotype reflecting a combination of all the molecular changes in the tumour, including germline and somatic genetic mutations and epigenetic changes, and tumour‐stroma interactions. An area for future research involves the integration of molecular subtype information with tumour morphology. There is particular interest in the possibility of incorporating digital pathology and artificial intelligence/machine learning approaches to develop tumour classification systems that integrate this wealth of morphological and molecular information [55, 56, 57]. This study demonstrates the importance of taking account of the information captured by morphological histological types as well as molecular subtypes in any future classifications.

## Conclusion

In this analysis of 838,776 women with breast cancer diagnosed in England during 1988–2016, we have shown distinct patterns in breast cancer incidence by tumour histological type. We have suggested where certain changes over time may be attributable to changing standards in reporting or associations with screening. We have also confirmed typical tumour characteristics for different histological types. This report will be of interest to epidemiologists studying patterns of incidence of breast cancer histological types. It may also assist clinical pathologists by providing reliable national data on reporting trends over time for local benchmarking. The variation in typical tumour characteristics between histological types also highlights the biological distinctiveness of these morphologically defined groups, supporting the value of classifications that capture tumour microscopic appearance in addition to molecular changes.

## Author contributions statement

CT, SCD, KG, JP, JB, DD and PM contributed to the study conceptualisation and design. JP, PM, JB, KG and DD contributed to the data collection and collation. JP, PM and SCD contributed to the data analysis. JP, SCD, PM, DD, CT and KG participated in data interpretation and manuscript preparation. All authors were involved in editing the paper and had final approval of the submitted and published versions.

## Supporting information


**Figure S1.** Composition of the study population among women diagnosed with invasive breast cancer in England during January 1988 to December 2016
**Figure S2.** Histological types of invasive breast carcinoma
**Text S1.** Explanatory note regarding breast cancer histopathology classification
**Table S1.** Summary of the changes in breast cancer classification over time, according to WHO Classification Editions
**Table S2.** ICD‐O3 codes for histological types of invasive breast cancer
**Table S3.** Approximations for breast cancer molecular subtypes using immunohistochemistry
**Table S4.** Age‐specific incidence rates (per 100,000 person‐years) and corresponding incidence rate ratios of 838,776 women diagnosed with invasive breast cancer in England during 1988–2016, according to each cancer histological type
**Table S5.** Age distribution (%) of 838,776 women diagnosed with invasive breast cancer in England during 1988–2016, according to cancer histological types
**Table S6.** Characteristics of 838,776 women diagnosed with invasive breast cancer in England during 1988–2016, grouped according to most common cancer histological types
**Table S7.** Characteristics of 10,616 woman diagnosed with rare histological types of breast cancer during 1988–2016
**Table S8.** Distribution of screen‐detection status in 838,776 women diagnosed with invasive breast cancer in England during 1988–2016, according to cancer histological types
**Table S9.** Age‐standardised incidence rates and rate ratios by calendar period of diagnosis by cancer histological type
**Table S10.** Characteristics of 246,477 women diagnosed with invasive breast cancer in England during 2010–2016, grouped according to most common cancer histological types
**Table S11.** Characteristics of 3923 women diagnosed with rare histological types of invasive breast cancer in England during 2010–2016
**Table S12.** Distribution of molecular subtype in 246,477 women diagnosed with invasive breast cancer in England during 2010–2016, according to cancer histological types
**Table S13.** Distribution of molecular subtype in 246,477 women diagnosed with invasive breast cancer in England during 2010–2016, according to cancer histological types, separately for age groups at diagnosis
**Table S14.** Distribution of molecular subtype in 246,477 women diagnosed with invasive breast cancer in England during 2010–2016, according to cancer histological types, separately for screen‐detection status
**Table S15.** Characteristics of 198,455 women diagnosed with ductal (no special type) carcinoma in England during 2010–2016, according to molecular subtype
**Figure S3.** Percentage of molecular subtype within each histological type for 246,477 women diagnosed during 2010–2016, split by age group at diagnosis
**Figure S4.** Percentage of molecular subtype within each histological type for 246,477 women diagnosed during 2010–2016, split by screen‐detection status

## Data Availability

De‐personalised study data may be made available on request to accredited researchers who submit a proposal that is approved by NHS England's Data Access Request Service (DARS).
