# Supplementary material for: Histological types of invasive breast cancer in 830,000 women diagnosed in England during 1988–2016
Source: J Pathol Clin Res. 2025 Sep 18;11(5):e70043. doi: 10.1002/2056-4538.70043 (PMC12445123; doi:10.1002/2056-4538.70043)

Histological types of invasive breast cancer in 830,000 women diagnosed in England during 1988–2016

J Probert *et al*. *J Pathol Clin Res* https://doi.org/10.1002/2056-4538.70043

Supplementary Material

Index

Methods

Figure S1	Composition of the study population among women diagnosed with invasive breast cancer in England during January 1988 to December 2016	4
Figure S2	Histological types of invasive breast carcinoma	5
Text S1	Explanatory note regarding breast cancer histopathology classification	8
Table S1	Summary of the changes in breast cancer classification over time, according to WHO Classification Editions	9
Table S2	ICD-O3 codes for histological types of invasive breast cancer	11
Table S3	Approximations for breast cancer molecular subtypes using immunohistochemistry	12

Results: Supplementary Tables

Table S4	Age-specific incidence rates (per 100,000 person-years) and corresponding incidence rate ratios of 838,776 women diagnosed with invasive breast cancer in England during 1988-2016, according to each cancer histological type (see Figure 1)	14
Table S5	Age distribution (%) of 838,776 women diagnosed with invasive breast cancer in England during 1988-2016, according to cancer histological types	16
Table S6	Characteristics of 838,776 women diagnosed with invasive breast cancer in England during 1988-2016, grouped according to most common cancer histological types	17
Table S7	Characteristics of 10,616 woman diagnosed with rare histological types of breast cancer during 1988-2016	18
Table S8	Distribution of screen-detection status in 838,776 women diagnosed with invasive breast cancer in England during 1988-2016, according to cancer histological types (see Figure 2)	19
Tables S9	Age-standardised incidence rates and rate ratios by calendar period of diagnosis by cancer histological type (see Figure 3)	20
Table S9i	Ductal carcinomas (NST, no special type)	20
Table S9ii	Lobular carcinomas	21
Table S9iii	Carcinomas of unspecified type	22

Table S9iv	Mucinous carcinomas	23
Table S9v	Tubular carcinomas	24
Table S9vi	Medullary carcinomas	25
Table S9vii	Metaplastic carcinomas	26
Table S9viii	Micropapillary carcinomas	27
Table S9ix	Papillary carcinomas	28
Table S9x	Apocrine carcinomas	29
Table S9xi	Cribriform carcinomas	30
Table S9xii	Other carcinomas	31
Table S9xiii	Adenoid cystic carcinomas	32
Table S9xiv	Neuroendocrine carcinomas	33
Table S9xv	Inflammatory carcinomas	34

Table S10	Characteristics of 246,477 women diagnosed with invasive breast cancer in England during 2010–2016, grouped according to most common cancer histological types	35
Table S11	Characteristics of 3923 women diagnosed with rare histological types of invasive breast cancer in England during 2010-2016	36
Table S12	Distribution of molecular subtype in 246,477 women diagnosed with invasive breast cancer in England during 2010–2016, according to cancer histological types (see Figure 5)	37
Table S13	Distribution of molecular subtype in 246,477 women diagnosed with invasive breast cancer in England during 2010–2016, according to cancer histological types, separately for age groups at diagnosis	38
Table S14	Distribution of molecular subtype in 246,477 women diagnosed with invasive breast cancer in England during 2010–2016, according to cancer histological types, separately for screen-detection status	39
Table S15	Characteristics of 198,455 women diagnosed with ductal (no special type) carcinoma in England during 2010–2016, according to molecular subtype	41

Results: Supplementary Figures

Figure S3	Percentage of molecular subtype within each histological type for 246,477 women diagnosed during 2010–2016, split by age group at diagnosis	43
Figure S4	Percentage of molecular subtype within each histological type for 246,477 women diagnosed during 2010–2016, split by screen-detection status	44

Methods

Figure S1: Composition of the study population among women diagnosed with invasive breast cancer in England during January 1988 to December 2016

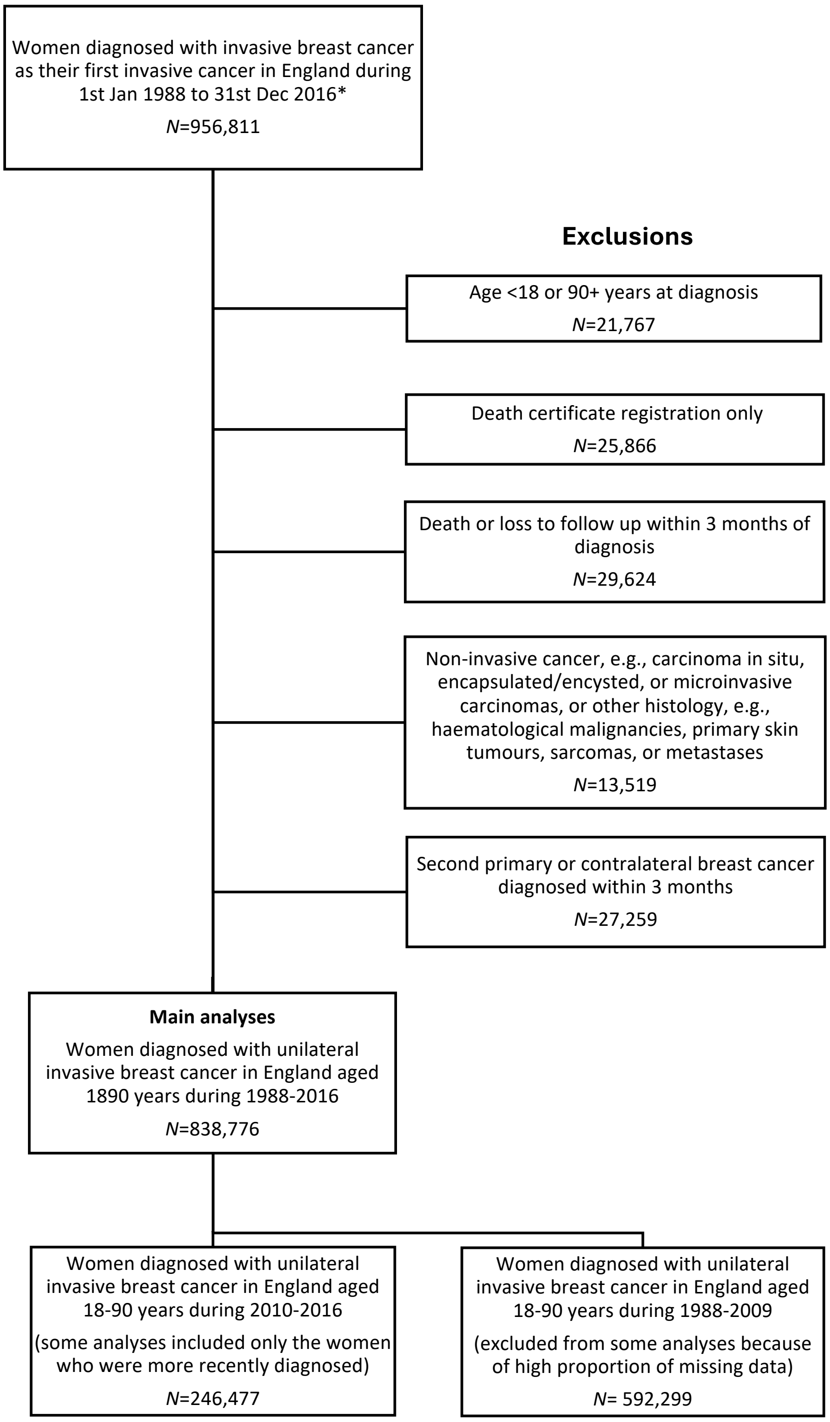

*Women were excluded if they had a prior invasive cancer at any other site, but were not excluded for prior non-invasive cancer.

Figure S2: Histological types of invasive breast carcinoma*

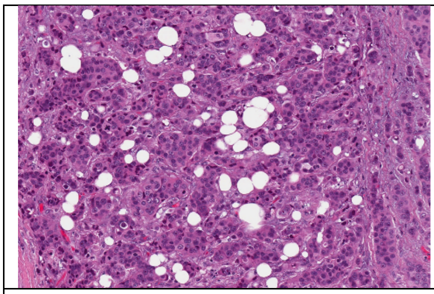

Ductal (NST)

A high-grade ductal carcinoma with poor tubule formation, large pleomorphic nuclei, and frequent mitoses.

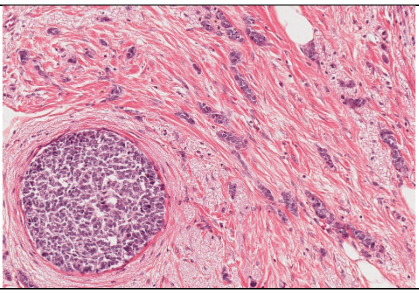

Lobular

A discohesive carcinoma with a ‘single filing’ pattern of infiltration and a nodule of associated lobular carcinoma in situ.

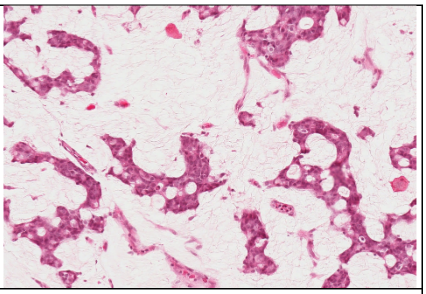

Mucinous

Small islands of tumour within large pools of pale-staining mucin.

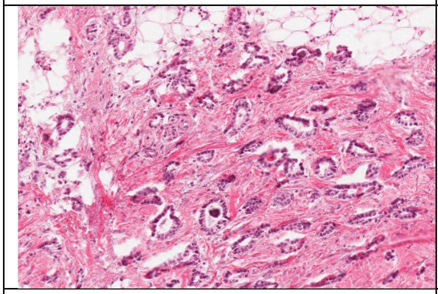

Tubular

Small infiltrating tubules with low-grade nuclear features.

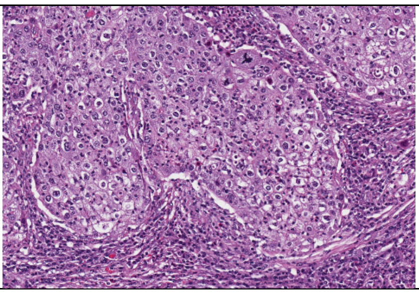

Medullary

Solid sheets of tumour cells with high-grade nuclear features and atypical mitoses, surrounded by a prominent lymphocytic response.

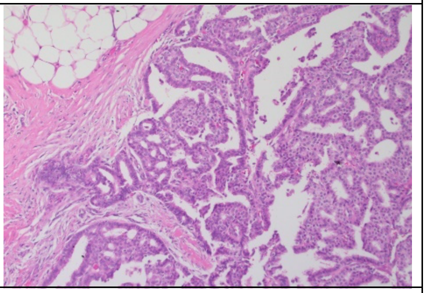

Papillary

Fibrovascular cores covered by neoplastic epithelium without myoepithelial cells, and showing invasion into the surrounding stroma.

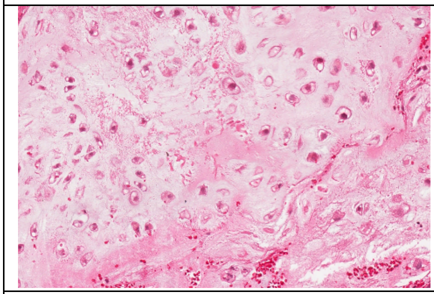

Metaplastic

The carcinoma in this case shows extensive metaplastic chondroid (cartilaginous) differentiation.

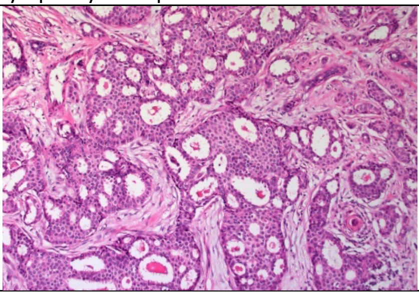

Cribriform

Invasive islands of epithelial cells forming cribriform (sieve-like) structures.

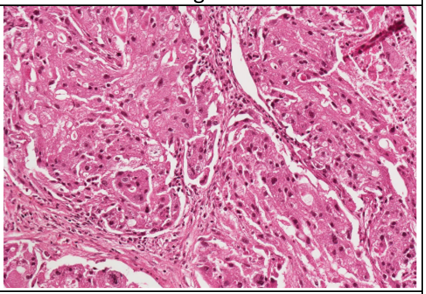

Apocrine

Tumour cells with abundant eosinophilic cytoplasm.

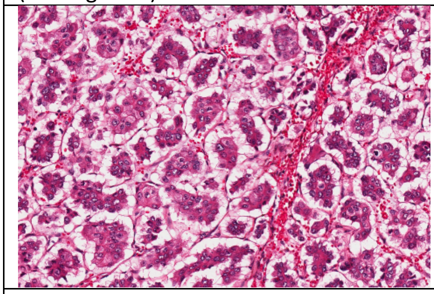

Micropapillary

A tumour composed entirely of small, fairly-uniform, papillary structures.

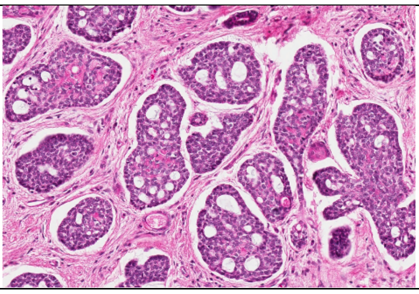

Adenoid cystic

Small, rounded nests of tumour with a pseudoglandular cribriform appearance.

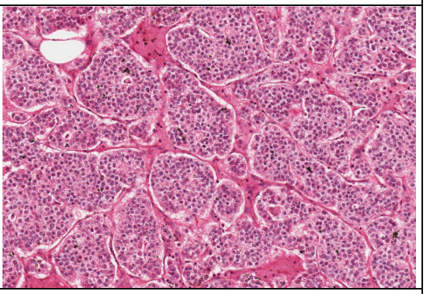

Neuroendocrine

Organoid nests and sheets of tumour cells with small round nuclei and stippled chromatin.

*Selected key morphological features are noted for each histological type

See following two pages for notes

Notes:

This figure illustrates some of the varied morphological features between the different histological types of invasive breast cancer featured in this paper. Please see the current World Health Organization Classification of Breast Tumours for more detailed descriptions, diagnostic criteria, and histological images (https://publications.iarc.fr/Book-And-Report-Series/Who-Classification-Of-Tumours/Breast-Tumours-2019). Inflammatory breast cancer is not shown as this is a clinical presentation rather than a true histological type. 'Other' and 'Unspecified' histological types are also not shown, as these will encompass a range of diagnoses.

Image sources:

Unless otherwise specified, images are used with permission from Juan Rosai's Collection of Surgical Pathology Seminars, and were taken at x20 magnification. Links are provided either to www.rosaicollection.org or https://rosaicollection.net or both (some images were taken via the former website, but this was temporarily offline at the time of manuscript submission).

Ductal carcinoma
https://www.rosaicollection.org/casedetails.cfm?c=14980
https://rosai.secondslide.com/view/sem1187/sem1187-case8.svs

Lobular carcinoma
https://www.rosaicollection.org/casedetails.cfm?c=5440
https://rosai.secondslide.com/view/sem235/sem235-case1.svs

Mucinous carcinoma
https://www.rosaicollection.org/casedetails.cfm?c=5088
https://rosai.secondslide.com/view/sem537/sem537-case5.svs

Tubular carcinoma
https://www.rosaicollection.org/casedetails.cfm?c=18862
https://rosai.secondslide.com/view/sem1475/sem1475-case4.svs

Medullary carcinoma
https://www.rosaicollection.org/casedetails.cfm?c=521
https://rosai.secondslide.com/view/sem1133/sem1133-case7.svs

Papillary carcinoma
https://librepathology.org/wiki/File:Breast_PapillaryCarcinoma_Invasive_MP_SNP.jpg
Source: Image of invasive papillary carcinoma from the Libre Pathology online resource. Author: Sarahkayb. This file is licensed under the Creative Commons Attribution-Share Alike 4.0 International license (https://creativecommons.org/licenses/by-sa/4.0/deed.en).

Metaplastic carcinoma
https://www.rosaicollection.org/casedetails.cfm?c=19092
https://rosai.secondslide.com/view/sem1484/sem1484-case23.svs

Cribriform carcinoma
https://librepathology.org/wiki/File:Breast_CribriformCarcinoma_MP_SNP.jpg
Source: Image of invasive cribriform carcinoma from the Libre Pathology online resource. Author: Sarahkayb. This file is licensed under the Creative Commons Attribution-Share Alike 4.0 International license (https://creativecommons.org/licenses/by-sa/4.0/deed.en).

Apocrine carcinoma
https://www.rosaicollection.org/casedetails.cfm?c=4942
https://rosai.secondslide.com/view/sem538/sem538-case9.svs

Micropapillary carcinoma
https://www.rosaicollection.org/casedetails.cfm?c=2797

Adenoid cystic carcinoma
https://www.rosaicollection.org/casedetails.cfm?c=4979

Neuroendocrine carcinoma
https://rosai.secondslide.com/view/sem578/sem578-case12.svs
Please note: The image used is from what would previously have been classified as a 'carcinoid tumour', and would now be considered a 'well-differentiated neuroendocrine tumour', within the spectrum of neuroendocrine neoplasms, reflecting the changing diagnostic criteria for these entities.

Text S1: Explanatory note regarding breast cancer histopathology classification

All cases in this study were classified as breast tumours by site/topography (i.e. ICD-9 code 174 or ICD-10 code C50). The main internationally-accepted classification for breast cancer histological subtypes is the World Health Organization (WHO) classification of breast tumours, which is revised periodically.[1] Over the 29-year period covered by this study (1988-2016), there were 3 editions of the WHO classification in use, published in 1981 (2nd Edition), 2003 (3rd Edition), and 2012 (4th Edition). Another edition was published in 2019 (5th Edition). Over the course of these editions, the classification system changed: some new diagnostic entities were defined (e.g. when evidence emerged of specific morphological or molecular features that are predictive of clinical behaviour), while other entities could be merged together. See **Table S1** for a summary of how the WHO classification has changed for the breast cancer histological types discussed in this study.

Data collection in cancer registries may use text descriptions of histological types, but will also use standardised codes. In the UK (and many other countries, though not all), this coding is typically based on the International Classification of Diseases for Oncology (ICD-O).[2] These codes are also referenced in the WHO tumour classification system, so that each diagnostic entity described will also have the relevant ICD-O code. The ICD-O coding system is also revised every few years, but these revisions are often not in sync with the WHO tumour pathology classification revisions. Of note, the ICD-O coding system covers all histopathological codes for neoplasms (i.e. including benign, malignant, and intermediate entities, and across all organ systems), whereas each organ system is covered by a different WHO tumour pathology classification book, each of which has a slightly differently-timed schedule of editions. In general, the meaning of ICD-O codes is fairly consistent between editions. When a new histopathological entity is defined, it will typically also receive a new ICD-O code. However, there are occasions when the meaning of an ICD-O code may change from one edition to another, for example with a pre-existing code being reassigned to a new entity. Over the 29-year period of this study, the ICD-O coding classification changed from the First Edition (published 1976), to the Second Edition ICD-O-2 (published 1990), to the Third Edition ICD-O-3 (published 2000, with edition 3.1 published in 2013, and edition 3.2 published in 2019).[3]

Given the above complexities of changing classification systems, in this study we have taken the pragmatic decision to attempt to map all histopathology text descriptions and ICD-O codes to what was the most recent WHO breast cancer pathology classification at the end of the study period (i.e. the edition published in 2012). ICD-O codes have been assigned to what was deemed to be the most likely histopathological entity. In the majority of cases, these codes will correspond to those listed in the WHO breast cancer pathology classification. However, where an ICD-O code was not specifically listed under an entity in the WHO breast tumour classification, it was reviewed by a pathologist (KG), and a judgement made as to which entity the code most closely corresponded to. **Table S2** shows the details of the histological types of invasive breast cancer used in this study, and the corresponding ICD-O codes of the cases within those groups.

---

[1] For an overview of the WHO classification of tumours, see: https://publications.iarc.fr/Book-And-Report-Series/Who-Classification-Of-Tumours [Accessed 22/03/2024]. For the 5th Edition of the Breast Cancer classification, see: https://publications.iarc.fr/Book-And-Report-Series/Who-Classification-Of-Tumours/Breast-Tumours-2019 [Accessed 22/03/2024].

[2] For a detailed history of the ICD-O classification, see: https://web.archive.org/web/20051115102100/http://training.seer.cancer.gov/module_coding_primary/unit01_hist_bkgrnd01.html [Accessed 22/03/2024].

[3] For details of ICD-O-3 revisions, see: https://www.who.int/standards/classifications/other-classifications/international-classification-of-diseases-for-oncology [Accessed 22/03/2024].

Table S1: Summary of the changes in breast cancer classification over time, according to WHO Classification Editions

	World Health Organization Classification Edition (year published)			
**Histological type**	**1981[1]**	**2003[2]**	**2012[3]**	**2019[4]**
Adenoid cystic carcinoma	✓	✓	✓	✓
Apocrine carcinoma	✓	✓	✓ 'Carcinoma with apocrine differentiation'	✓ 'Carcinoma with apocrine differentiation' Note: Desirable diagnostic criteria include immunoprofile (ER-negative, PR-negative, androgen receptor positive).
Ductal carcinoma NST (no special type)	✓ 'Invasive ductal carcinoma'	✓ 'Invasive ductal carcinoma, NOS'	✓ 'Invasive carcinoma of no special type (NST)' with several variants	✓ 'Invasive breast carcinoma of no special type (NST)'
Cribriform carcinoma	× Does not appear in the classification	✓ **New entity** Accompanying notes mention the need to differentiate from cribriform DCIS and adenoid cystic carcinoma.	✓	✓ Note: Diagnostic criteria include immunoprofile (ER-positive, HER2-negative).
Inflammatory carcinoma	✓ Mentioned as a clinical entity	✓	✓ Mentioned as a clinical pattern	✓ Mentioned as a clinical presentation
Lobular carcinoma	✓ 'Invasive lobular carcinoma'	✓	✓ 'Invasive lobular carcinoma'	✓ 'Invasive lobular carcinoma'
Medullary carcinoma	✓	✓	✓ 'Carcinoma with medullary features' including Medullary carcinoma, Atypical medullary carcinoma, and Invasive carcinoma NST with medullary features	✓ 'Medullary pattern' is now considered to be a morphological pattern of invasive breast carcinoma NST (ductal).
Metaplastic carcinoma	✓ 'Carcinoma with metaplasia'	✓	✓ 'Metaplastic carcinoma of no special type' and variants	✓ 'Metaplastic carcinoma'

(table continued)

(table continued)

	World Health Organization Classification Edition (year published)			
**Histological type**	**1981[1]**	**2003[2]**	**2012[3]**	**2019[4]**
Micropapillary carcinoma	✗<br>Does not appear in the classification	✓ **New entity**<br>'Invasive micropapillary carcinoma'	✓<br>'Invasive micropapillary carcinoma'	✓<br>'Invasive micropapillary carcinoma'
Mucinous carcinoma	✓	✓<br>'Mucin producing carcinomas'	✓	✓
Neuroendocrine carcinoma/ tumour	✗<br>Not named in main classification, though 'carcinomas with carcinoid features' (a type of neuroendocrine tumour) are mentioned under 'Other' (p.21)	✓ **New entity**<br>'Neuroendocrine tumours'	✓<br>'Carcinoma with neuroendocrine features' including variants depending on whether well- or poorly- differentiated	✓<br>Shifting classification, including 'Neuroendocrine tumour', 'Neuroendocrine carcinoma', and mixed invasive carcinoma.
Papillary carcinoma	✓	✓<br>'Invasive papillary carcinoma'	✓<br>'Invasive papillary carcinoma'	✓<br>Shifting classification, including 'Encapsulated papillary carcinoma', 'Solid papillary carcinoma (invasive)', and 'Invasive papillary carcinoma'
Tubular carcinoma	✓	✓	✓	✓<br>Note: Diagnostic criteria include immunoprofile (ER-positive and HER2-negative).

[1] World Health Organization. Histological typing of breast tumours, 2nd ed. Geneva: World Health Organization, 1981.
[2] Devilee P, Tavassoli F. Pathology and genetics of tumours of the breast and female genital organs, 3rd ed. Lyon: International Agency for Research on Cancer, 2003.
[3] Lakhani SR, Ellis IO, Schnitt SJ, Tan PH, van de Vijver MJ. WHO Classification of Tumours of the Breast, 4th ed. France: International Agency for Research on Cancer, 2012.
[4] WHO Classification of Tumours Editorial Board. WHO Classification of Tumours: Breast Tumours, 5th ed. France: International Agency for Research on Cancer; 2019.
✓ Entity named in this edition of the WHO Breast Cancer Classification.
✗ Entity not named in this edition of the WHO Breast Cancer Classification.

Table S2: ICD-O3 codes for histological types of invasive breast cancer

Invasive breast cancer histological type*	ICD-O3 codes†
Adenoid cystic	82003
Apocrine	84013, 85733
Ductal	80223, 80343, 80353, 80393, 81413, 82313, 85003, 85143, 85213, 85223, 85233, 85413
Cribriform	82013
Inflammatory	85303
Lobular	85203, 85243
Medullary	85103, 85123, 85133
Metaplastic	80303, 80313, 80323, 80333, 80523, 80703, 80713, 80723, 80743, 85603, 85703, 85713, 85723, 85753, 89803, 89823
Micropapillary	85073
Mucinous	84803, 84813, 84903
Neuroendocrine	80133, 80413, 82401, 82403, 82413, 82433, 82453, 82463, 82493, 85743
Papillary	80503, 82603, 83443, 85033, 85053
Tubular	82113
Other	80823, 81473, 81903, 83103, 83143, 83153, 83903, 84003, 84103, 84303, 85023, 85083, 85093, 85503, 85623, 89403, 89833
Unspecified	80003, 80013, 80033, 80043, 80103, 80113, 80123, 80203, 80213, 81403, 81453, 82033, 82053, 82303, 82553, 83233, 85293

*Ordered alphabetically for specific types, then 'Other' and 'Unspecified'

†Codes from the International Classification of Diseases for Oncology, Third Edition

Table S3: Approximations for breast cancer molecular subtypes using immunohistochemistry

Intrinsic molecular subtype	St. Gallen surrogate definition*	Surrogate definition using grade instead of Ki-67 (as used in this study)
Luminal A	ER and/or PR positive,<br>HER2 negative,<br>Ki-67 low	ER and/or PR positive,<br>HER2 negative,<br>grade 1 or 2
Luminal B (HER2 negative)	ER and/or PR positive,<br>HER2 negative,<br>Ki-67 high	ER and/or PR positive,<br>HER2 negative,<br>grade 3
Luminal B (HER2 positive)	ER and/or PR positive,<br>HER2 over-expressed or amplified,<br>any Ki-67	ER and/or PR positive,<br>HER2 positive,<br>any grade
HER2 positive (non-luminal)	ER and PR negative,<br>HER2 over-expressed or amplified,<br>any Ki-67	ER and PR negative,<br>HER2 positive,<br>any grade
Triple-negative (basal-like)	Negative for ER, PR, and HER2,<br>any Ki-67	Negative for ER, PR, and HER2,<br>any grade

*Based on: Goldhirsch A, Wood WC, Coates AS, Gelber RD, Thurlimann B, Senn HJ, et al. Strategies for subtypes--dealing with the diversity of breast cancer: highlights of the St. Gallen International Expert Consensus on the Primary Therapy of Early Breast Cancer 2011. Ann Oncol. 2011;22(8):1736-47.

Results: Supplementary Tables

Table S4: Age-specific incidence rates (per 100,000 person-years) and corresponding incidence rate ratios of 838,776 women diagnosed with invasive breast cancer in England during 1988-2016, according to each cancer histological type (see Figure 1)†‡

Cancer histological type	Age (years)	Number of cases	Age-specific incidence rate per 100,000 PY (95% CI)	Incidence rate ratio (95% CI)
Ductal ($p < 0.0001$)	18-39	38,786	17.3 (17.1, 17.5)	0.09 (0.09, 0.09)
	40-49	110,643	109.0 (108.4, 109.7)	0.6 (0.6, 0.6)
	50-64	240,493	190.7 (189.9, 191.4)	1.0 (1.0, 1.0)*
	65-70	84,196	196.4 (195.0, 197.7)	1.0 (1.0, 1.0)
	71-79	87,578	168.1 (167.0, 169.2)	0.9 (0.9, 0.9)
	80-89	53,002	156.7 (155.3, 158.0)	0.8 (0.8, 0.8)
Lobular ($p < 0.0001$)	18-39	2213	0.99 (0.95, 1.03)	0.04 (0.03, 0.04)
	40-49	13,207	13.0 (12.8, 13.2)	0.5 (0.5, 0.5)
	50-64	35,054	27.8 (27.5, 28.1)	1.0 (1.0, 1.0)*
	65-70	14,243	33.2 (32.7, 33.8)	1.2 (1.2, 1.2)
	71-79	15,493	29.7 (29.3, 30.2)	1.1 (1.1, 1.1)
	80-89	9818	29.0 (28.5, 29.6)	1.0 (1.0, 1.1)
Unspecified ($p < 0.0001$)	18-39	4093	1.8 (1.8, 1.9)	0.1 (0.1, 0.1)
	40-49	10,414	10.3 (10.1, 10.5)	0.6 (0.6, 0.6)
	50-64	20,747	16.4 (16.2, 16.7)	1.0 (1.0, 1.0)*
	65-70	9614	22.4 (22.0, 22.9)	1.4 (1.3, 1.4)
	71-79	19,250	37.0 (36.4, 37.5)	2.2 (2.2, 2.3)
	80-89	22,542	66.6 (65.8, 67.5)	4.1 (4.0, 4.1)
Mucinous ($p < 0.0001$)	18-39	400	0.18 (0.16, 0.20)	0.06 (0.05, 0.07)
	40-49	1511	1.5 (1.4, 1.6)	0.5 (0.5, 0.5)
	50-64	3800	3.0 (2.9, 3.1)	1.0 (1.0, 1.0)*
	65-70	2510	5.9 (5.6, 6.1)	1.9 (1.9, 2.0)
	71-79	4264	8.2 (7.9, 8.4)	2.7 (2.6, 2.8)
	80-89	3876	11.5 (11.1, 11.8)	3.8 (3.7, 3.9)
Tubular ($p < 0.0001$)	18-39	372	0.17 (0.15, 0.18)	0.02 (0.02, 0.02)
	40-49	2603	2.6 (2.5, 2.7)	0.3 (0.3, 0.4)
	50-64	9377	7.4 (7.3, 7.6)	1.0 (1.0, 1.0)*
	65-70	2157	5.0 (4.8, 5.2)	0.7 (0.6, 0.7)
	71-79	1204	2.3 (2.2, 2.4)	0.3 (0.3, 0.3)
	80-89	405	1.2 (1.1, 1.3)	0.2 (0.1, 0.2)
Medullary ($p < 0.0001$)	18-39	703	0.31 (0.29, 0.34)	0.2 (0.2, 0.3)
	40-49	1048	1.0 (1.0, 1.1)	0.8 (0.8, 0.9)
	50-64	1582	1.3 (1.2, 1.3)	1.0 (1.0, 1.1)*
	65-70	433	1.0 (0.9, 1.1)	0.8 (0.7, 0.9)
	71-79	378	0.73 (0.66, 0.80)	0.6 (0.5, 0.6)
	80-89	151	0.45 (0.38, 0.52)	0.4 (0.3, 0.4)
Metaplastic ($p < 0.0001$)	18-39	117	0.05 (0.04, 0.06)	0.09 (0.08, 0.11)
	40-49	289	0.28 (0.25, 0.32)	0.5 (0.5, 0.6)
	50-64	699	0.55 (0.51, 0.60)	1.0 (0.9, 1.1)*
	65-70	329	0.77 (0.69, 0.85)	1.4 (1.2, 1.5)
	71-79	452	0.87 (0.79, 0.95)	1.6 (1.4, 1.7)
	80-89	364	1.1 (1.0, 1.2)	1.9 (1.8, 2.2)

(table continued)

(table continued)

Micropapillary ($p < 0.0001$)	18-39	36	0.02 (0.01, 0.02)	0.07 (0.05, 0.10)
	40-49	103	0.10 (0.08, 0.12)	0.5 (0.4, 0.6)
	50-64	274	0.22 (0.19, 0.24)	1.0 (0.9, 1.1)*
	65-70	138	0.32 (0.27, 0.38)	1.5 (1.3, 1.8)
	71-79	137	0.26 (0.22, 0.31)	1.2 (1.0, 1.4)
	80-89	75	0.22 (0.18, 0.28)	1.0 (0.8, 1.3)
Papillary ($p < 0.0001$)	18-39	86	0.04 (0.03, 0.05)	0.06 (0.05, 0.07)
	40-49	253	0.25 (0.22, 0.28)	0.4 (0.3, 0.4)
	50-64	841	0.67 (0.62, 0.71)	1.0 (0.9, 1.1)*
	65-70	598	1.4 (1.3, 1.5)	2.1 (1.9, 2.3)
	71-79	880	1.7 (1.6, 1.8)	2.5 (2.4, 2.7)
	80-89	637	1.9 (1.7, 2.0)	2.8 (2.6, 3.1)
Apocrine ($p < 0.0001$)	18-39	28	0.01 (0.01, 0.02)	0.05 (0.03, 0.07)
	40-49	91	0.09 (0.07, 0.11)	0.4 (0.3, 0.4)
	50-64	323	0.26 (0.23, 0.29)	1.0 (0.9, 1.1)*
	65-70	186	0.43 (0.38, 0.50)	1.7 (1.5, 2.0)
	71-79	172	0.33 (0.28, 0.38)	1.3 (1.1, 1.5)
	80-89	151	0.45 (0.38, 0.52)	1.7 (1.5, 2.0)
Cribriform ($p < 0.0001$)	18-39	61	0.03 (0.02, 0.03)	0.05 (0.04, 0.07)
	40-49	286	0.28 (0.25, 0.32)	0.6 (0.5, 0.6)
	50-64	630	0.50 (0.46, 0.54)	1.0 (0.9, 1.1)*
	65-70	233	0.54 (0.48, 0.62)	1.1 (1.0, 1.2)
	71-79	250	0.48 (0.42, 0.54)	1.0 (0.8, 1.1)
	80-89	157	0.46 (0.40, 0.54)	0.9 (0.8, 1.1)
Other ($p < 0.0001$)	18-39	41	0.02 (0.01, 0.02)	0.2 (0.1, 0.3)
	40-49	69	0.07 (0.05, 0.09)	0.8 (0.6, 1.0)
	50-64	114	0.09 (0.08, 0.11)	1.0 (0.8, 1.2)*
	65-70	66	0.15 (0.12, 0.20)	1.7 (1.3, 2.2)
	71-79	72	0.14 (0.11, 0.17)	1.5 (1.2, 1.9)
	80-89	52	0.15 (0.12, 0.20)	1.7 (1.3, 2.2)
Adenoid Cystic ($p < 0.0001$)	18-39	26	0.01 (0.01, 0.02)	0.07 (0.05, 0.11)
	40-49	78	0.08 (0.06, 0.10)	0.5 (0.4, 0.6)
	50-64	201	0.16 (0.14, 0.18)	1.0 (0.9, 1.1)*
	65-70	80	0.19 (0.15, 0.23)	1.2 (0.9, 1.5)
	71-79	73	0.14 (0.11, 0.18)	0.9 (0.7, 1.1)
	80-89	51	0.15 (0.11, 0.20)	0.9 (0.7, 1.2)
Neuroendocrine ($p < 0.0001$)	18-39	8	0.004 (0.002, 0.007)	0.03 (0.02, 0.07)
	40-49	47	0.05 (0.03, 0.06)	0.4 (0.3, 0.6)
	50-64	131	0.10 (0.09, 0.12)	1.0 (0.8, 1.2)*
	65-70	64	0.15 (0.12, 0.19)	1.4 (1.1, 1.8)
	71-79	116	0.22 (0.19, 0.27)	2.1 (1.8, 2.6)
	80-89	97	0.29 (0.23, 0.35)	2.8 (2.3, 3.4)
Inflammatory ($p < 0.0001$)	18-39	47	0.02 (0.02, 0.03)	0.2 (0.1, 0.3)
	40-49	78	0.08 (0.06, 0.10)	0.7 (0.6, 0.9)
	50-64	136	0.11 (0.09, 0.13)	1.0 (0.8, 1.2)*
	65-70	36	0.08 (0.06, 0.12)	0.8 (0.6, 1.1)
	71-79	34	0.07 (0.05, 0.09)	0.6 (0.4, 0.8)
	80-89	23	0.07 (0.05, 0.10)	0.6 (0.4, 0.9)

*Reference category. †Histological types ordered by total number of cases. *p* values are for trend.

‡ The number of decimal places reported for incidence rates and rate ratios is dependent on the magnitude of the point estimate. For incidence rates (per 100,000), one decimal place is given for rates of at least 1, while two decimal places are given for estimates between 0.1 and 1 and for rates below 0.1 only one significant figure is reported. Incidence rate ratios are reported to 1 decimal place unless they are less than 0.1 in which case they are reported to 1 significant figure.

Table S5: Age distribution (%) of 838,776 women diagnosed with invasive breast cancer in England during 1988-2016, according to cancer histological types.

Cancer histological type*	Age distribution (%) within each cancer type						Total %	Total number of women
	18-39	40-49	50-64	65-70	71-79	80-89		
**Ductal**	6.3	18.0	39.1	13.7	14.2	8.7	100	614,698
**Lobular**	2.5	14.7	38.9	15.8	17.2	10.9	100	90,028
**Unspecified**	4.7	12.0	23.9	11.1	22.2	26.1	100	86,660
**Mucinous**	2.4	9.2	23.2	15.3	26.1	23.8	100	16,361
**Tubular**	2.3	16.1	58.2	13.4	7.5	2.5	100	16,118
**Medullary**	16.4	24.4	36.8	10.1	8.8	3.5	100	4295
**Papillary**	2.6	7.7	25.5	18.1	26.7	19.4	100	3295
**Metaplastic**	5.2	12.8	31.1	14.6	20.1	16.2	100	2250
**Cribriform**	3.8	17.7	39.0	14.4	15.5	9.6	100	1617
**Apocrine**	2.9	9.6	34.0	19.6	18.1	15.8	100	951
**Micropapillary**	4.7	13.5	35.9	18.1	18.0	9.8	100	763
**Adenoid Cystic**	5.1	15.3	39.5	15.7	14.3	10.1	100	509
**Neuroendocrine**	1.7	10.2	28.3	13.8	25.1	20.9	100	463
**Other**	9.9	16.7	27.5	15.9	17.4	12.6	100	414
**Inflammatory**	13.3	22.0	38.4	10.2	9.6	6.5	100	354
**All types**	**5.6**	**16.8**	**37.5**	**13.7**	**15.5**	**10.9**	**100**	**-**
**Total number of women**	**47,017**	**140,720**	**314,402**	**114,883**	**130,353**	**91,401**	**-**	**838,776**

*Cancer histological types are ordered by the number of cases

Table S6: Characteristics of 838,776 women diagnosed with invasive breast cancer in England during 1988-2016, grouped according to most common cancer histological types. See **Table S7** for rare histological types.

Characteristic*	Histological type, %							Total %	Total number of women
	Ductal	Lobular	Mucinous	Tubular	Medullary	Rare subtypes†	Unspecified		
**Diagnosis year** ($p < 0.001$)									
1988-1992	57.9	8.7	1.7	1.7	1.2	1.1	27.7	100	109,333
1993-1999	66.1	10.4	1.9	2.4	0.8	1.1	17.3	100	177,357
2000-2004	74.8	11.1	2.0	2.1	0.5	1.0	8.5	100	146,519
2005-2009	79.2	11.0	2.0	1.8	0.3	1.3	4.4	100	159,090
2010-2016	80.5	11.5	2.0	1.6	0.2	1.6	2.6	100	246,477
**Age at diagnosis** ($p < 0.001$)									
18-39	82.5	4.7	0.9	0.8	1.5	1.0	8.6	100	47,017
40-49	78.6	9.4	1.1	1.8	0.7	0.9	7.5	100	140,720
50-64	76.5	11.1	1.2	3.0	0.5	1.1	6.6	100	314,402
65-70	73.3	12.4	2.2	1.9	0.4	1.5	8.3	100	114,883
71-79	67.2	11.9	3.3	0.9	0.3	1.7	14.7	100	130,353
80-89	58.0	10.7	4.2	0.4	0.2	1.8	24.7	100	91,401
**Cancer screen detected** ($p < 0.001$)									
Eligible: screen detected	79.9	11.1	1.6	4.2	0.2	1.0	2.0	100	154,991
Eligible: not screen detected (interval cancer)	77.8	13.5	1.3	1.2	0.5	1.5	4.2	100	63,160
Eligible: not screen detected (other)	73.4	11.1	1.2	2.1	0.6	1.2	10.4	100	158,366
Not eligible for screening	70.4	10.1	2.4	1.2	0.6	1.3	14.0	100	462,259
**Breast cancer laterality** ($p < 0.001$)									
Left	74.4	11.1	2.0	1.9	0.5	1.3	8.8	100	414,061
Right	75.0	10.7	1.9	2.0	0.5	1.2	8.7	100	387,451
Unknown	42.4	7.2	1.4	1.1	0.9	0.9	46.1	100	37,264
**Index of multiple deprivation** ($p < 0.001$)									
<20%, least deprived	74.2	11.5	1.8	2.0	0.5	1.2	8.8	100	185,110
20-39%	73.3	11.3	1.9	2.0	0.5	1.2	9.8	100	187,217
40-59%	73.0	10.9	1.9	1.9	0.5	1.3	10.5	100	176,250
60-79%	72.7	10.2	2.1	1.8	0.5	1.3	11.4	100	158,148
80+%, most deprived	73.1	9.2	2.2	1.8	0.5	1.3	11.9	100	132,051
**Geographical region** ($p < 0.001$)									
Eastern	73.8	12.1	2.0	1.0	0.5	1.5	9.1	100	90,072
North West	75.9	10.4	2.0	1.6	0.4	1.1	8.6	100	110,110
Northern & Yorkshire	74.1	10.1	2.0	2.3	0.5	1.3	9.7	100	110,581
Oxford	73.5	11.8	2.0	2.2	0.8	1.4	8.3	100	47,979
South West	73.2	11.3	1.9	1.9	0.4	1.2	10.1	100	126,683
Thames	74.0	9.9	1.9	1.4	0.4	1.1	11.3	100	180,583
Trent	68.4	9.9	2.0	3.2	0.5	1.3	14.7	100	81,042
West Midlands	71.6	11.6	2.1	2.6	0.6	1.5	10.0	100	91,726
**Total %**	**73.3**	**10.7**	**2.0**	**1.9**	**0.5**	**1.3**	**10.3**	**100**	**-**
**Total number of women**	**614,698**	**90,028**	**16,361**	**16,118**	**4,295**	**10,616**	**86,660**	**-**	**838,776**

* p values are for χ^2 tests of independence between known characteristic values and common histological types

† all subtypes that are not mentioned in the table (including "Other")

Table S7: Characteristics of 10,616 women diagnosed with rare histological types of breast cancer during 1988-2016. See **Table S6** for common histological types.

**Characteristic***	**Rare histological type, %**									**Total % of rare subtypes**	**Total number of women**
	**Papillary**	**Metaplastic**	**Cribriform**	**Apocrine**	**Micro-papillary**	**Adenoid cystic**	**Neuro-endocrine**	**Inflammatory**	**Other**		
**Diagnosis year** (*p* < 0.001)											
1988-1992	0.37	0.18	0.29	0.03	<0.01	0.04	0.08	0.02	0.09	1.10	109,333
1993-1999	0.36	0.18	0.31	0.04	<0.01	0.05	0.05	0.07	0.04	1.11	177,357
2000-2004	0.38	0.18	0.18	0.09	0.01	0.06	0.03	0.06	0.03	1.02	146,519
2005-2009	0.49	0.28	0.13	0.14	0.04	0.07	0.05	0.03	0.04	1.28	159,090
2010-2016	0.37	0.42	0.11	0.20	0.28	0.07	0.07	0.03	0.06	1.59	246,477
**Age at diagnosis** (*p* < 0.001)											
18-39	0.18	0.25	0.13	0.06	0.08	0.06	0.02	0.10	0.09	0.96	47,017
40-49	0.18	0.21	0.20	0.06	0.07	0.06	0.03	0.06	0.05	0.92	140,720
50-64	0.27	0.22	0.20	0.10	0.09	0.06	0.04	0.04	0.04	1.07	314,402
65-70	0.52	0.29	0.20	0.16	0.12	0.07	0.06	0.03	0.06	1.51	114,883
71-79	0.68	0.35	0.19	0.13	0.11	0.06	0.09	0.03	0.06	1.68	130,353
80-89	0.70	0.40	0.17	0.17	0.08	0.06	0.11	0.03	0.06	1.76	91,401
**Cancer screen detected** (*p* < 0.001)											
Eligible: screen detected	0.28	0.11	0.20	0.14	0.15	0.07	0.03	0.01	0.03	1.02	154,991
Eligible: not screen detected (interval cancer)	0.37	0.47	0.16	0.18	0.12	0.07	0.06	0.08	0.04	1.55	63,160
Eligible: not screen detected (other)	0.31	0.28	0.20	0.09	0.06	0.07	0.05	0.06	0.04	1.16	158,366
Not eligible for screening	0.46	0.29	0.19	0.10	0.08	0.06	0.06	0.04	0.06	1.35	462,259
**Breast cancer laterality** (*p* = 0.30)											
Left	0.41	0.28	0.19	0.13	0.10	0.06	0.05	0.04	0.05	1.31	414,061
Right	0.39	0.26	0.19	0.11	0.09	0.06	0.05	0.05	0.05	1.25	387,451
Unknown	0.30	0.19	0.19	0.02	0.01	0.05	0.08	0.03	0.04	0.91	37,264
**Index of multiple deprivation** (*p* = 0.17)											
<20%, least deprived	0.38	0.25	0.18	0.11	0.09	0.06	0.05	0.04	0.05	1.22	185,110
20-39%	0.40	0.24	0.19	0.11	0.08	0.06	0.06	0.04	0.05	1.23	187,217
40-59%	0.39	0.27	0.20	0.10	0.09	0.06	0.06	0.05	0.06	1.26	176,250
60-79%	0.40	0.29	0.21	0.12	0.09	0.05	0.05	0.04	0.05	1.30	158,148
80+%, most deprived	0.41	0.30	0.19	0.13	0.10	0.08	0.06	0.04	0.04	1.34	132,051
**Geographical region** (*p* < 0.001)											
Eastern	0.46	0.32	0.22	0.11	0.11	0.05	0.09	0.05	0.06	1.49	90,072
North West	0.34	0.27	0.19	0.11	0.04	0.05	0.06	0.02	0.04	1.11	110,110
Northern & Yorkshire	0.35	0.33	0.16	0.16	0.13	0.06	0.06	0.07	0.03	1.34	110,581
Oxford	0.45	0.21	0.25	0.18	0.10	0.06	0.06	0.01	0.05	1.38	47,979
South West	0.42	0.23	0.13	0.09	0.04	0.07	0.04	0.04	0.07	1.15	126,683
Thames	0.33	0.20	0.17	0.09	0.07	0.06	0.06	0.06	0.05	1.09	180,583
Trent	0.45	0.30	0.19	0.13	0.12	0.06	0.03	0.02	0.04	1.33	81,042
West Midlands	0.46	0.32	0.31	0.10	0.16	0.07	0.05	0.03	0.05	1.54	91,726
**Total %**	**0.39**	**0.27**	**0.19**	**0.11**	**0.09**	**0.06**	**0.06**	**0.04**	**0.05**	**1.27**	**-**
**Total number of women**	**3295**	**2250**	**1617**	**951**	**763**	**509**	**463**	**354**	**414**	**-**	**838,776**

* *p* values are for χ² tests of independence between known characteristic values and rare histological types

Table S8: Distribution of screen-detection status in 838,776 women diagnosed with invasive breast cancer in England during 1988-2016, according to cancer histological types (see Figure 2)

Cancer histological type*	Distribution of screen detection status, n (%)					Total %	Total number of women
	Eligible: screen detected	Eligible: not screen detected (interval cancer)	Eligible: not screen detected (other)	Below regular screening age	Above regular screening age		
Tubular	6585 (40.9)	762 (4.7)	3300 (20.5)	2975 (18.5)	2496 (15.4)	100	16,118
Micropapillary	239 (31.3)	74 (9.7)	97 (12.7)	139 (18.2)	214 (28.1)	100	763
Apocrine	217 (22.8)	111 (11.7)	146 (15.4)	119 (12.5)	358 (37.6)	100	951
Ductal	123,915 (20.2)	49,154 (8.0)	116,215 (18.9)	149,429 (24.3)	175,985 (28.6)	100	614,698
Adenoid Cystic	102 (20.0)	44 (8.6)	105 (20.6)	104 (20.4)	154 (30.4)	100	509
Lobular	17,183 (19.1)	8506 (9.4)	17,551 (19.5)	15,420 (17.1)	31,368 (34.9)	100	90,028
Cribriform	303 (18.7)	101 (6.2)	317 (19.6)	347 (21.5)	549 (34.0)	100	1617
Mucinous	2426 (14.8)	832 (5.1)	1902 (11.6)	1911 (11.7)	9290 (56.8)	100	16,361
Papillary	430 (13.1)	235 (7.1)	492 (14.9)	339 (10.3)	1799 (54.6)	100	3295
Other	53 (12.8)	25 (6.0)	70 (16.9)	110 (26.6)	156 (37.7)	100	414
Neuroendocrine	53 (11.4)	39 (8.4)	73 (15.8)	55 (11.9)	243 (52.5)	100	463
Medullary	336 (7.8)	328 (7.6)	1015 (23.6)	1751 (40.8)	865 (20.2)	100	4295
Metaplastic	175 (7.8)	300 (13.3)	440 (19.6)	406 (18.0)	929 (41.3)	100	2250
Unspecified	2964 (3.4)	2601 (3.0)	16,550 (19.1)	14,507 (16.7)	50,038 (57.8)	100	86,660
Inflammatory	10 (2.8)	48 (13.6)	93 (26.3)	125 (35.3)	78 (22.0)	100	354
**Total %**	**18.5**	**7.5**	**18.9**	**22.4**	**32.7**	**100**	**-**
**Total number of women**	**154,991**	**63,160**	**158,366**	**187,737**	**274,522**	**-**	**838,776**

*Cancer histological types are ordered by the percentage of screen-detected cancers

Tables S9: Age-standardised incidence rates and rate ratios by calendar period of diagnosis by cancer histological type (see Figure 3)*

Table S9i: Age-standardised incidence rates and rate ratios by calendar period of diagnosis for ductal carcinomas (NST, no special type)

Cancer histological type	Calendar year of diagnosis	Number of cases	Crude incidence rate per 100,000 PY	Age-standardised incidence rate per 100,000 PY (95% CI)	Age-standardised incidence rate ratio (95% CI)
Ductal	1988	10,223	54.1	57.5 (56.4,58.7)	1.0 [Ref]
	1989	11,771	62.0	66.1 (64.9,67.3)	1.1 (1.1,1.2)
	1990	12,874	67.6	72.7 (71.4,74.0)	1.3 (1.2,1.3)
	1991	13,906	72.7	78.7 (77.4,80.1)	1.4 (1.3,1.4)
	1992	14,544	76.0	81.7 (80.4,83.0)	1.4 (1.4,1.5)
	1993	14,256	74.4	79.4 (78.1,80.7)	1.4 (1.3,1.4)
	1994	14,594	76.1	80.5 (79.2,81.9)	1.4 (1.4,1.4)
	1995	15,686	81.7	86.1 (84.7,87.4)	1.5 (1.5,1.5)
	1996	16,513	85.9	89.9 (88.5,91.3)	1.6 (1.5,1.6)
	1997	17,816	92.5	96.2 (94.8,97.6)	1.7 (1.6,1.7)
	1998	18,837	97.5	101.0 (99.5,102.4)	1.8 (1.7,1.8)
	1999	19,575	100.8	104.1 (102.7,105.6)	1.8 (1.8,1.9)
	2000	20,183	103.4	106.2 (104.7,107.7)	1.8 (1.8,1.9)
	2001	20,820	106.2	108.5 (107.1,110.0)	1.9 (1.8,1.9)
	2002	21,684	110.1	112.5 (111.0,114.1)	2.0 (1.9,2.0)
	2003	23,269	117.5	119.7 (118.2,121.3)	2.1 (2.0,2.1)
	2004	23,642	118.6	120.8 (119.3,122.4)	2.1 (2.1,2.2)
	2005	24,239	120.4	123.1 (121.5,124.7)	2.1 (2.1,2.2)
	2006	24,683	121.6	124.1 (122.5,125.7)	2.2 (2.1,2.2)
	2007	25,005	122.2	124.5 (122.9,126.0)	2.2 (2.1,2.2)
	2008	25,838	125.1	126.8 (125.2,128.4)	2.2 (2.2,2.3)
	2009	26,285	126.3	127.3 (125.7,128.8)	2.2 (2.2,2.3)
	2010	27,112	129.2	129.7 (128.1,131.3)	2.3 (2.2,2.3)
	2011	26,805	126.8	127.0 (125.5,128.5)	2.2 (2.2,2.3)
	2012	27,643	130.0	129.3 (127.7,130.8)	2.2 (2.2,2.3)
	2013	28,748	134.4	133.0 (131.5,134.6)	2.3 (2.3,2.4)
	2014	29,659	137.6	135.7 (134.2,137.3)	2.4 (2.3,2.4)
	2015	29,198	134.4	131.9 (130.4,133.4)	2.3 (2.2,2.3)
	2016	29,290	133.9	131.0 (129.5,132.5)	2.3 (2.2,2.3)

* The incidence of common and rare subtypes can vary over several orders of magnitude, especially when subdivided by factors such as age. Therefore, the number of decimal places reported for both crude and age-standardised incidence rates and rate ratios is dependent on the magnitude of the point estimate. For incidence rates (per 100,000), one decimal place is given for rates of at least 1, while two decimal places are given for estimates between 0.1 and 1 and for rates below 0.1 only one significant figure is reported. Incidence rate ratios are reported to 1 decimal place unless they are less than 0.1 in which case they are reported to 1 significant figure.

Table S9ii: Age-standardised incidence rates and rate ratios by calendar period of diagnosis for lobular carcinomas

Cancer histological type	Calendar year of diagnosis	Number of cases	Crude incidence rate per 100,000 PY	Age-standardised incidence rate per 100,000 PY (95% CI)	Age-standardised incidence rate ratio (95% CI)
Lobular	1988	1496	7.9	8.4 (8.0,8.9)	1.0 [Ref]
	1989	1760	9.3	9.9 (9.5,10.4)	1.2 (1.1,1.3)
	1990	1911	10.0	10.9 (10.4,11.4)	1.3 (1.2,1.4)
	1991	2053	10.7	11.7 (11.2,12.2)	1.4 (1.3,1.5)
	1992	2280	11.9	12.8 (12.3,13.4)	1.5 (1.4,1.6)
	1993	2164	11.3	12.1 (11.6,12.6)	1.4 (1.3,1.5)
	1994	2179	11.4	12.1 (11.6,12.6)	1.4 (1.3,1.5)
	1995	2426	12.6	13.4 (12.9,14.0)	1.6 (1.5,1.7)
	1996	2623	13.7	14.4 (13.9,15.0)	1.7 (1.6,1.8)
	1997	2881	15.0	15.7 (15.1,16.2)	1.9 (1.7,2.0)
	1998	3093	16.0	16.8 (16.2,17.4)	2.0 (1.9,2.1)
	1999	3014	15.5	16.1 (15.6,16.7)	1.9 (1.8,2.0)
	2000	3184	16.3	16.9 (16.3,17.5)	2.0 (1.9,2.1)
	2001	3186	16.3	16.9 (16.3,17.4)	2.0 (1.9,2.1)
	2002	3268	16.6	17.2 (16.6,17.8)	2.0 (1.9,2.2)
	2003	3366	17.0	17.5 (16.9,18.1)	2.1 (2.0,2.2)
	2004	3300	16.6	17.0 (16.4,17.6)	2.0 (1.9,2.1)
	2005	3321	16.5	17.0 (16.5,17.6)	2.0 (1.9,2.2)
	2006	3335	16.4	16.9 (16.3,17.5)	2.0 (1.9,2.1)
	2007	3415	16.7	17.1 (16.6,17.7)	2.0 (1.9,2.2)
	2008	3762	18.2	18.6 (18.0,19.2)	2.2 (2.1,2.3)
	2009	3610	17.3	17.5 (16.9,18.1)	2.1 (2.0,2.2)
	2010	3667	17.5	17.7 (17.1,18.3)	2.1 (2.0,2.2)
	2011	3820	18.1	18.2 (17.7,18.8)	2.2 (2.0,2.3)
	2012	3804	17.9	17.8 (17.2,18.4)	2.1 (2.0,2.2)
	2013	4225	19.7	19.6 (19.0,20.2)	2.3 (2.2,2.5)
	2014	4270	19.8	19.6 (19.0,20.2)	2.3 (2.2,2.5)
	2015	4420	20.3	20.0 (19.4,20.6)	2.4 (2.2,2.5)
	2016	4195	19.2	18.7 (18.2,19.3)	2.2 (2.1,2.4)

Table S9iii: Age-standardised incidence rates and rate ratios by calendar period of diagnosis for carcinomas of unspecified type

Cancer histological type	Calendar year of diagnosis	Number of cases	Crude incidence rate per 100,000 PY	Age-standardised incidence rate per 100,000 PY (95% CI)	Age-standardised incidence rate ratio (95% CI)
Unspecified	1988	7018	37.1	38.3 (37.4,39.2)	1.0 [Ref]
	1989	6510	34.3	35.2 (34.3,36.1)	0.9 (0.9,0.9)
	1990	5944	31.2	31.9 (31.1,32.7)	0.8 (0.8,0.9)
	1991	5604	29.3	29.9 (29.2,30.7)	0.8 (0.8,0.8)
	1992	5282	27.6	27.9 (27.1,28.7)	0.7 (0.7,0.8)
	1993	4792	25.0	25.1 (24.3,25.8)	0.7 (0.6,0.7)
	1994	4599	24.0	23.9 (23.2,24.6)	0.6 (0.6,0.6)
	1995	4844	25.2	25.0 (24.3,25.7)	0.7 (0.6,0.7)
	1996	4366	22.7	22.2 (21.5,22.9)	0.6 (0.6,0.6)
	1997	4402	22.9	22.3 (21.7,23.0)	0.6 (0.6,0.6)
	1998	3724	19.3	18.7 (18.1,19.3)	0.5 (0.5,0.5)
	1999	3980	20.5	20.1 (19.5,20.7)	0.5 (0.5,0.5)
	2000	3298	16.9	16.5 (15.9,17.0)	0.4 (0.4,0.4)
	2001	2956	15.1	14.7 (14.1,15.2)	0.4 (0.4,0.4)
	2002	2374	12.0	11.7 (11.3,12.2)	0.3 (0.3,0.3)
	2003	1921	9.7	9.4 (9.0,9.9)	0.2 (0.2,0.3)
	2004	1746	8.8	8.6 (8.2,9.0)	0.2 (0.2,0.2)
	2005	1661	8.3	8.1 (7.7,8.5)	0.2 (0.2,0.2)
	2006	1469	7.2	7.1 (6.7,7.5)	0.2 (0.2,0.2)
	2007	1293	6.3	6.1 (5.8,6.5)	0.2 (0.1,0.2)
	2008	1363	6.6	6.4 (6.1,6.8)	0.2 (0.2,0.2)
	2009	1196	5.7	5.5 (5.2,5.9)	0.1 (0.1,0.2)
	2010	1196	5.7	5.5 (5.2,5.8)	0.1 (0.1,0.2)
	2011	1248	5.9	5.8 (5.4,6.1)	0.2 (0.1,0.2)
	2012	907	4.3	4.1 (3.9,4.4)	0.1 (0.1,0.1)
	2013	879	4.1	4.0 (3.7,4.2)	0.1 (0.1,0.1)
	2014	807	3.7	3.6 (3.3,3.8)	0.1 (0.1,0.1)
	2015	724	3.3	3.2 (3.0,3.5)	0.1 (0.1,0.1)
	2016	557	2.5	2.4 (2.2,2.7)	0.1 (0.1,0.1)

Table S9iv: Age-standardised incidence rates and rate ratios by calendar period of diagnosis for mucinous carcinomas

Cancer histological type	Calendar year of diagnosis	Number of cases	Crude incidence rate per 100,000 PY	Age-standardised incidence rate per 100,000 PY (95% CI)	Age-standardised incidence rate ratio (95% CI)
Mucinous	1988	349	1.8	1.9 (1.7,2.1)	1.0 [Ref]
	1989	326	1.7	1.7 (1.6,1.9)	0.9 (0.8,1.1)
	1990	365	1.9	2.0 (1.8,2.2)	1.0 (0.9,1.2)
	1991	393	2.1	2.1 (1.9,2.3)	1.1 (1.0,1.3)
	1992	371	1.9	2.0 (1.8,2.2)	1.1 (0.9,1.2)
	1993	374	2.0	2.0 (1.8,2.2)	1.1 (0.9,1.2)
	1994	456	2.4	2.4 (2.2,2.6)	1.3 (1.1,1.5)
	1995	450	2.3	2.4 (2.2,2.6)	1.3 (1.1,1.5)
	1996	486	2.5	2.6 (2.3,2.8)	1.4 (1.2,1.6)
	1997	508	2.6	2.7 (2.4,2.9)	1.4 (1.2,1.6)
	1998	522	2.7	2.7 (2.5,3.0)	1.5 (1.3,1.7)
	1999	568	2.9	2.9 (2.7,3.2)	1.6 (1.4,1.8)
	2000	568	2.9	2.9 (2.7,3.2)	1.6 (1.4,1.8)
	2001	593	3.0	3.0 (2.8,3.3)	1.6 (1.4,1.8)
	2002	571	2.9	2.9 (2.7,3.2)	1.6 (1.4,1.8)
	2003	626	3.2	3.2 (2.9,3.5)	1.7 (1.5,2.0)
	2004	619	3.1	3.1 (2.9,3.4)	1.7 (1.5,1.9)
	2005	634	3.2	3.2 (2.9,3.4)	1.7 (1.5,1.9)
	2006	655	3.2	3.2 (3.0,3.5)	1.7 (1.5,2.0)
	2007	602	2.9	2.9 (2.7,3.2)	1.6 (1.4,1.8)
	2008	716	3.5	3.4 (3.2,3.7)	1.8 (1.6,2.1)
	2009	649	3.1	3.1 (2.9,3.4)	1.7 (1.4,1.9)
	2010	674	3.2	3.2 (2.9,3.4)	1.7 (1.5,1.9)
	2011	672	3.2	3.2 (2.9,3.4)	1.7 (1.5,1.9)
	2012	718	3.4	3.3 (3.1,3.6)	1.8 (1.6,2.0)
	2013	697	3.3	3.2 (3.0,3.4)	1.7 (1.5,1.9)
	2014	742	3.4	3.3 (3.1,3.6)	1.8 (1.6,2.0)
	2015	724	3.3	3.2 (3.0,3.5)	1.7 (1.5,2.0)
	2016	733	3.4	3.2 (3.0,3.5)	1.7 (1.5,2.0)

Table S9v: Age-standardised incidence rates and rate ratios by calendar period of diagnosis for tubular carcinomas

Cancer histological type	Calendar year of diagnosis	Number of cases	Crude incidence rate per 100,000 PY	Age-standardised incidence rate per 100,000 PY (95% CI)	Age-standardised incidence rate ratio (95% CI)
Tubular	1988	180	0.95	1.0 (0.9,1.2)	1.0 [Ref]
	1989	289	1.5	1.7 (1.5,1.9)	1.6 (1.3,2.0)
	1990	371	1.9	2.2 (1.9,2.4)	2.1 (1.8,2.5)
	1991	461	2.4	2.7 (2.5,3.0)	2.6 (2.2,3.2)
	1992	570	3.0	3.3 (3.1,3.6)	3.3 (2.7,3.9)
	1993	520	2.7	3.0 (2.8,3.3)	3.0 (2.5,3.5)
	1994	549	2.9	3.1 (2.9,3.4)	3.1 (2.6,3.6)
	1995	586	3.1	3.3 (3.1,3.6)	3.3 (2.7,3.9)
	1996	578	3.0	3.3 (3.0,3.5)	3.2 (2.7,3.8)
	1997	693	3.6	3.8 (3.6,4.1)	3.7 (3.2,4.4)
	1998	668	3.5	3.7 (3.4,4.0)	3.6 (3.0,4.2)
	1999	663	3.4	3.6 (3.3,3.9)	3.5 (3.0,4.1)
	2000	668	3.4	3.6 (3.3,3.8)	3.5 (2.9,4.1)
	2001	627	3.2	3.3 (3.1,3.6)	3.2 (2.7,3.9)
	2002	521	2.6	2.8 (2.5,3.0)	2.7 (2.3,3.2)
	2003	673	3.4	3.6 (3.3,3.9)	3.5 (2.9,4.1)
	2004	658	3.3	3.5 (3.2,3.7)	3.4 (2.9,4.0)
	2005	638	3.2	3.3 (3.1,3.6)	3.2 (2.7,3.9)
	2006	611	3.0	3.2 (2.9,3.4)	3.1 (2.6,3.7)
	2007	523	2.6	2.7 (2.4,2.9)	2.6 (2.2,3.1)
	2008	541	2.6	2.7 (2.5,3.0)	2.7 (2.2,3.2)
	2009	537	2.6	2.7 (2.5,2.9)	2.6 (2.2,3.1)
	2010	506	2.4	2.5 (2.3,2.7)	2.4 (2.0,2.9)
	2011	600	2.8	2.9 (2.7,3.1)	2.8 (2.4,3.3)
	2012	650	3.1	3.1 (2.8,3.3)	3.0 (2.5,3.6)
	2013	579	2.7	2.7 (2.5,2.9)	2.6 (2.2,3.1)
	2014	557	2.6	2.5 (2.3,2.8)	2.5 (2.1,3.0)
	2015	540	2.5	2.4 (2.2,2.7)	2.4 (2.0,2.8)
	2016	561	2.6	2.5 (2.3,2.7)	2.4 (2.1,2.9)

Table S9vi: Age-standardised incidence rates and rate ratios by calendar period of diagnosis for medullary carcinomas

Cancer histological type	Calendar year of diagnosis	Number of cases	Crude incidence rate per 100,000 PY	Age-standardised incidence rate per 100,000 PY (95% CI)	Age-standardised incidence rate ratio (95% CI)
Medullary	1988	250	1.3	1.4 (1.3,1.6)	1.0 [Ref]
	1989	285	1.5	1.6 (1.4,1.8)	1.1 (0.9,1.3)
	1990	264	1.4	1.5 (1.3,1.7)	1.0 (0.9,1.2)
	1991	220	1.2	1.2 (1.1,1.4)	0.9 (0.7,1.0)
	1992	256	1.3	1.4 (1.2,1.6)	1.0 (0.8,1.2)
	1993	225	1.2	1.2 (1.1,1.4)	0.9 (0.7,1.0)
	1994	249	1.3	1.4 (1.2,1.6)	1.0 (0.8,1.1)
	1995	194	1.0	1.0 (0.9,1.2)	0.7 (0.6,0.9)
	1996	219	1.1	1.2 (1.0,1.3)	0.8 (0.7,1.0)
	1997	199	1.0	1.1 (0.9,1.2)	0.8 (0.6,0.9)
	1998	181	0.94	0.94 (0.81,1.09)	0.7 (0.5,0.8)
	1999	137	0.71	0.72 (0.61,0.86)	0.5 (0.4,0.6)
	2000	195	1.00	1.0 (0.9,1.2)	0.7 (0.6,0.9)
	2001	152	0.78	0.78 (0.66,0.91)	0.5 (0.4,0.7)
	2002	123	0.62	0.64 (0.53,0.76)	0.4 (0.4,0.6)
	2003	124	0.63	0.62 (0.52,0.74)	0.4 (0.3,0.5)
	2004	116	0.58	0.57 (0.47,0.68)	0.4 (0.3,0.5)
	2005	115	0.57	0.58 (0.48,0.70)	0.4 (0.3,0.5)
	2006	97	0.48	0.47 (0.38,0.57)	0.3 (0.3,0.4)
	2007	119	0.58	0.58 (0.48,0.70)	0.4 (0.3,0.5)
	2008	91	0.44	0.44 (0.35,0.54)	0.3 (0.2,0.4)
	2009	57	0.27	0.27 (0.21,0.35)	0.2 (0.1,0.3)
	2010	80	0.38	0.38 (0.30,0.47)	0.3 (0.2,0.3)
	2011	65	0.31	0.31 (0.24,0.39)	0.2 (0.2,0.3)
	2012	65	0.31	0.30 (0.23,0.38)	0.2 (0.2,0.3)
	2013	55	0.26	0.25 (0.19,0.33)	0.2 (0.1,0.2)
	2014	44	0.20	0.20 (0.15,0.27)	0.1 (0.1,0.2)
	2015	59	0.27	0.27 (0.20,0.34)	0.2 (0.1,0.2)
	2016	59	0.27	0.27 (0.20,0.35)	0.2 (0.1,0.3)

Table S9vii: Age-standardised incidence rates and rate ratios by calendar period of diagnosis for metaplastic carcinomas

Cancer histological type	Calendar year of diagnosis	Number of cases	Crude incidence rate per 100,000 PY	Age-standardised incidence rate per 100,000 PY (95% CI)	Age-standardised incidence rate ratio (95% CI)
Metaplastic	1988	41	0.22	0.23 (0.16,0.31)	1.0 [Ref]
	1989	42	0.22	0.23 (0.17,0.32)	1.0 (0.6,1.6)
	1990	41	0.22	0.23 (0.16,0.31)	1.0 (0.6,1.6)
	1991	44	0.23	0.23 (0.17,0.32)	1.0 (0.7,1.6)
	1992	34	0.18	0.19 (0.13,0.27)	0.8 (0.5,1.3)
	1993	42	0.22	0.23 (0.16,0.31)	1.0 (0.6,1.6)
	1994	38	0.20	0.20 (0.14,0.28)	0.9 (0.6,1.4)
	1995	46	0.24	0.24 (0.17,0.32)	1.1 (0.7,1.6)
	1996	51	0.27	0.26 (0.20,0.35)	1.2 (0.7,1.8)
	1997	46	0.24	0.25 (0.19,0.34)	1.1 (0.7,1.7)
	1998	44	0.23	0.23 (0.17,0.31)	1.0 (0.7,1.6)
	1999	47	0.24	0.26 (0.19,0.35)	1.1 (0.7,1.8)
	2000	37	0.19	0.19 (0.14,0.27)	0.9 (0.5,1.4)
	2001	50	0.26	0.25 (0.19,0.34)	1.1 (0.7,1.7)
	2002	49	0.25	0.25 (0.19,0.33)	1.1 (0.7,1.7)
	2003	62	0.31	0.32 (0.25,0.41)	1.4 (0.9,2.1)
	2004	62	0.31	0.31 (0.24,0.40)	1.4 (0.9,2.1)
	2005	71	0.35	0.36 (0.28,0.46)	1.6 (1.1,2.4)
	2006	64	0.32	0.33 (0.25,0.42)	1.5 (1.0,2.2)
	2007	100	0.49	0.50 (0.41,0.61)	2.2 (1.5,3.3)
	2008	103	0.50	0.51 (0.41,0.62)	2.2 (1.5,3.3)
	2009	111	0.53	0.54 (0.45,0.66)	2.4 (1.7,3.5)
	2010	107	0.51	0.51 (0.42,0.62)	2.2 (1.5,3.3)
	2011	117	0.55	0.55 (0.46,0.66)	2.4 (1.7,3.6)
	2012	142	0.67	0.66 (0.55,0.78)	2.9 (2.0,4.2)
	2013	165	0.77	0.76 (0.65,0.89)	3.4 (2.4,4.9)
	2014	150	0.70	0.69 (0.58,0.81)	3.0 (2.1,4.4)
	2015	160	0.74	0.72 (0.61,0.84)	3.1 (2.2,4.6)
	2016	184	0.84	0.82 (0.71,0.95)	3.6 (2.6,5.2)

Table S9viii: Age-standardised incidence rates and rate ratios by calendar period of diagnosis for micropapillary carcinomas

Cancer histological type	Calendar year of diagnosis	Number of cases	Crude incidence rate per 100,000 PY	Age-standardised incidence rate per 100,000 PY (95% CI)	Age-standardised incidence rate ratio (95% CI)
Micropapillary	2003	2	0.01	0.01 (0.001,0.04)	1.0 [Ref]
	2004	6	0.03	0.03 (0.01,0.07)	2.8 (0.5,28.5)
	2005	7	0.03	0.04 (0.01,0.08)	3.2 (0.6,31.2)
	2006	13	0.06	0.06 (0.03,0.11)	5.6 (1.3,50.8)
	2007	8	0.04	0.04 (0.02,0.07)	3.1 (0.6,30.2)
	2008	15	0.07	0.07 (0.04,0.12)	6.2 (1.5,56.4)
	2009	26	0.12	0.13 (0.08,0.19)	10.8 (2.8,94.3)
	2010	30	0.14	0.15 (0.10,0.21)	12.5 (3.2,108.0)
	2011	53	0.25	0.26 (0.19,0.34)	22.1 (5.9,187.0)
	2012	76	0.36	0.36 (0.28,0.45)	31.1 (8.5,261.8)
	2013	94	0.44	0.44 (0.36,0.54)	37.9 (10.4,317.7)
	2014	136	0.63	0.63 (0.53,0.75)	54.4 (15.1,453.5)
	2015	135	0.62	0.61 (0.51,0.73)	52.7 (14.6,439.9)
	2016	161	0.74	0.72 (0.62,0.84)	62.3 (17.3,518.6)

Table S9ix: Age-standardised incidence rates and rate ratios by calendar period of diagnosis for papillary carcinomas

Cancer histological type	Calendar year of diagnosis	Number of cases	Crude incidence rate per 100,000 PY	Age-standardised incidence rate per 100,000 PY (95% CI)	Age-standardised incidence rate ratio (95% CI)
Papillary	1988	85	0.45	0.46 (0.36,0.57)	1.0 [Ref]
	1989	71	0.37	0.38 (0.30,0.48)	0.8 (0.6,1.2)
	1990	83	0.44	0.45 (0.36,0.56)	1.0 (0.7,1.4)
	1991	85	0.44	0.45 (0.36,0.56)	1.0 (0.7,1.3)
	1992	77	0.40	0.41 (0.33,0.52)	0.9 (0.7,1.2)
	1993	93	0.49	0.48 (0.39,0.59)	1.1 (0.8,1.4)
	1994	94	0.49	0.51 (0.41,0.62)	1.1 (0.8,1.5)
	1995	101	0.53	0.54 (0.44,0.65)	1.2 (0.9,1.6)
	1996	83	0.43	0.44 (0.35,0.54)	1.0 (0.7,1.3)
	1997	111	0.58	0.59 (0.49,0.71)	1.3 (1.0,1.7)
	1998	92	0.48	0.48 (0.39,0.59)	1.0 (0.8,1.4)
	1999	68	0.35	0.35 (0.27,0.45)	0.8 (0.6,1.1)
	2000	107	0.55	0.56 (0.46,0.68)	1.2 (0.9,1.7)
	2001	101	0.52	0.53 (0.43,0.65)	1.2 (0.9,1.6)
	2002	110	0.56	0.57 (0.47,0.69)	1.3 (0.9,1.7)
	2003	123	0.62	0.64 (0.53,0.76)	1.4 (1.0,1.9)
	2004	112	0.56	0.56 (0.46,0.68)	1.2 (0.9,1.7)
	2005	134	0.67	0.67 (0.56,0.80)	1.5 (1.1,2.0)
	2006	144	0.71	0.73 (0.61,0.86)	1.6 (1.2,2.1)
	2007	168	0.82	0.83 (0.71,0.97)	1.8 (1.4,2.4)
	2008	150	0.73	0.72 (0.61,0.85)	1.6 (1.2,2.1)
	2009	179	0.86	0.86 (0.74,1.00)	1.9 (1.4,2.5)
	2010	164	0.78	0.78 (0.66,0.91)	1.7 (1.3,2.2)
	2011	158	0.75	0.76 (0.65,0.89)	1.7 (1.3,2.2)
	2012	151	0.71	0.72 (0.61,0.84)	1.6 (1.2,2.1)
	2013	117	0.55	0.55 (0.46,0.66)	1.2 (0.9,1.6)
	2014	108	0.50	0.49 (0.40,0.60)	1.1 (0.8,1.5)
	2015	126	0.58	0.57 (0.48,0.68)	1.3 (0.9,1.7)
	2016	100	0.46	0.45 (0.37,0.55)	1.0 (0.7,1.3)

Table S9x: Age-standardised incidence rates and rate ratios by calendar period of diagnosis for apocrine carcinomas

Cancer histological type	Calendar year of diagnosis	Number of cases	Crude incidence rate per 100,000 PY	Age-standardised incidence rate per 100,000 PY (95% CI)	Age-standardised incidence rate ratio (95% CI)
Apocrine	1988	5	0.03	0.03 (0.01,0.07)	1.0 [Ref]
	1989	5	0.03	0.03 (0.01,0.07)	1.1 (0.2,4.6)
	1990	7	0.04	0.04 (0.01,0.08)	1.3 (0.4,5.2)
	1991	6	0.03	0.03 (0.01,0.07)	1.1 (0.3,4.7)
	1992	6	0.03	0.03 (0.01,0.07)	1.1 (0.3,4.8)
	1993	10	0.05	0.05 (0.02,0.10)	1.8 (0.6,6.9)
	1994	4	0.02	0.02 (0.01,0.06)	0.8 (0.2,3.7)
	1995	8	0.04	0.04 (0.02,0.08)	1.5 (0.4,5.9)
	1996	8	0.04	0.04 (0.02,0.08)	1.5 (0.4,5.9)
	1997	13	0.07	0.07 (0.04,0.12)	2.4 (0.8,8.6)
	1998	16	0.08	0.08 (0.05,0.13)	2.9 (1.0,10.2)
	1999	18	0.09	0.10 (0.06,0.16)	3.5 (1.3,12.2)
	2000	25	0.13	0.13 (0.09,0.20)	4.7 (1.8,15.9)
	2001	16	0.08	0.08 (0.05,0.13)	2.9 (1.0,10.2)
	2002	23	0.12	0.12 (0.08,0.19)	4.4 (1.6,14.8)
	2003	29	0.15	0.15 (0.10,0.22)	5.3 (2.0,17.7)
	2004	38	0.19	0.19 (0.14,0.27)	6.9 (2.7,22.4)
	2005	44	0.22	0.22 (0.16,0.29)	7.6 (3.0,24.6)
	2006	37	0.18	0.19 (0.14,0.27)	6.8 (2.7,22.3)
	2007	50	0.24	0.25 (0.18,0.33)	8.8 (3.5,28.3)
	2008	44	0.21	0.22 (0.16,0.30)	7.8 (3.1,25.2)
	2009	53	0.25	0.26 (0.20,0.34)	9.2 (3.7,29.6)
	2010	54	0.26	0.26 (0.20,0.34)	9.3 (3.7,29.8)
	2011	55	0.26	0.26 (0.19,0.34)	9.1 (3.7,29.4)
	2012	62	0.29	0.29 (0.22,0.38)	10.3 (4.2,33.0)
	2013	78	0.36	0.36 (0.28,0.45)	12.6 (5.2,40.0)
	2014	63	0.29	0.29 (0.22,0.37)	10.1 (4.1,32.4)
	2015	78	0.36	0.35 (0.27,0.43)	12.2 (5.0,38.9)
	2016	96	0.44	0.42 (0.34,0.52)	15.0 (6.2,47.3)

Table S9xi: Age-standardised incidence rates and rate ratios by calendar period of diagnosis for cribriform carcinomas

Cancer histological type	Calendar year of diagnosis	Number of cases	Crude incidence rate per 100,000 PY	Age-standardised incidence rate per 100,000 PY (95% CI)	Age-standardised incidence rate ratio (95% CI)
Cribriform	1988	37	0.20	0.21 (0.15,0.29)	1.0 [Ref]
	1989	53	0.28	0.29 (0.22,0.38)	1.4 (0.9,2.2)
	1990	73	0.38	0.41 (0.32,0.52)	2.0 (1.3,3.0)
	1991	68	0.36	0.38 (0.30,0.49)	1.8 (1.2,2.8)
	1992	91	0.48	0.51 (0.41,0.62)	2.4 (1.6,3.7)
	1993	102	0.53	0.58 (0.47,0.70)	2.8 (1.9,4.1)
	1994	84	0.44	0.46 (0.36,0.57)	2.2 (1.5,3.3)
	1995	76	0.40	0.42 (0.33,0.52)	2.0 (1.3,3.0)
	1996	93	0.48	0.51 (0.41,0.62)	2.4 (1.6,3.7)
	1997	73	0.38	0.39 (0.31,0.50)	1.9 (1.2,2.9)
	1998	70	0.36	0.37 (0.29,0.47)	1.8 (1.2,2.7)
	1999	59	0.30	0.31 (0.24,0.40)	1.5 (1.0,2.3)
	2000	64	0.33	0.33 (0.25,0.42)	1.6 (1.0,2.4)
	2001	71	0.36	0.37 (0.29,0.47)	1.8 (1.2,2.7)
	2002	50	0.25	0.26 (0.19,0.34)	1.2 (0.8,2.0)
	2003	42	0.21	0.23 (0.16,0.30)	1.1 (0.7,1.7)
	2004	39	0.20	0.20 (0.14,0.28)	1.0 (0.6,1.5)
	2005	46	0.23	0.23 (0.17,0.31)	1.1 (0.7,1.8)
	2006	48	0.24	0.24 (0.18,0.32)	1.2 (0.7,1.8)
	2007	37	0.18	0.19 (0.13,0.26)	0.9 (0.6,1.5)
	2008	30	0.15	0.15 (0.10,0.22)	0.7 (0.4,1.2)
	2009	40	0.19	0.20 (0.14,0.27)	0.9 (0.6,1.5)
	2010	27	0.13	0.12 (0.08,0.18)	0.6 (0.3,1.0)
	2011	47	0.22	0.22 (0.16,0.29)	1.0 (0.7,1.7)
	2012	40	0.19	0.19 (0.13,0.25)	0.9 (0.5,1.4)
	2013	37	0.17	0.17 (0.12,0.24)	0.8 (0.5,1.3)
	2014	35	0.16	0.16 (0.11,0.23)	0.8 (0.5,1.3)
	2015	46	0.21	0.21 (0.15,0.28)	1.0 (0.6,1.6)
	2016	39	0.18	0.18 (0.13,0.24)	0.8 (0.5,1.4)

Table S9xii: Age-standardised incidence rates and rate ratios by calendar period of diagnosis for other carcinomas

Cancer histological type	Calendar year of diagnosis	Number of cases	Crude incidence rate per 100,000 PY	Age-standardised incidence rate per 100,000 PY (95% CI)	Age-standardised incidence rate ratio (95% CI)
Other	1988	43	0.23	0.24 (0.17,0.32)	1.0 [Ref]
	1989	17	0.09	0.09 (0.05,0.15)	0.4 (0.2,0.7)
	1990	12	0.06	0.07 (0.03,0.12)	0.3 (0.1,0.5)
	1991	15	0.08	0.08 (0.05,0.14)	0.3 (0.2,0.6)
	1992	6	0.03	0.03 (0.01,0.07)	0.1 (0.0,0.3)
	1993	18	0.09	0.10 (0.06,0.15)	0.4 (0.2,0.7)
	1994	13	0.07	0.07 (0.04,0.12)	0.3 (0.1,0.6)
	1995	10	0.05	0.05 (0.03,0.10)	0.2 (0.1,0.5)
	1996	7	0.04	0.04 (0.02,0.08)	0.2 (0.1,0.4)
	1997	11	0.06	0.06 (0.03,0.11)	0.3 (0.1,0.5)
	1998	11	0.06	0.06 (0.03,0.11)	0.2 (0.1,0.5)
	1999	5	0.03	0.03 (0.01,0.06)	0.1 (0.0,0.3)
	2000	11	0.06	0.06 (0.03,0.11)	0.2 (0.1,0.5)
	2001	9	0.05	0.05 (0.02,0.09)	0.2 (0.1,0.4)
	2002	8	0.04	0.04 (0.02,0.08)	0.2 (0.1,0.4)
	2003	8	0.04	0.04 (0.02,0.08)	0.2 (0.1,0.3)
	2004	11	0.06	0.06 (0.03,0.10)	0.2 (0.1,0.5)
	2005	9	0.04	0.05 (0.02,0.09)	0.2 (0.1,0.4)
	2006	12	0.06	0.05 (0.03,0.10)	0.2 (0.1,0.5)
	2007	16	0.08	0.08 (0.05,0.13)	0.3 (0.2,0.6)
	2008	8	0.04	0.04 (0.02,0.08)	0.2 (0.1,0.4)
	2009	14	0.07	0.06 (0.03,0.11)	0.3 (0.1,0.5)
	2010	10	0.05	0.04 (0.02,0.08)	0.2 (0.1,0.4)
	2011	12	0.06	0.05 (0.03,0.10)	0.2 (0.1,0.5)
	2012	12	0.06	0.05 (0.03,0.10)	0.2 (0.1,0.5)
	2013	14	0.07	0.06 (0.03,0.10)	0.3 (0.1,0.5)
	2014	10	0.05	0.05 (0.02,0.09)	0.2 (0.1,0.4)
	2015	30	0.14	0.14 (0.09,0.19)	0.6 (0.3,0.9)
	2016	52	0.24	0.23 (0.17,0.31)	1.0 (0.6,1.5)

Table S9xiii: Age-standardised incidence rates and rate ratios by calendar period of diagnosis for adenoid cystic carcinomas

Cancer histological type	Calendar year of diagnosis	Number of cases	Crude incidence rate per 100,000 PY	Age-standardised incidence rate per 100,000 PY (95% CI)	Age-standardised incidence rate ratio (95% CI)
Adenoid Cystic	1988	9	0.05	0.05 (0.02,0.10)	1.0 [Ref]
	1989	12	0.06	0.07 (0.03,0.11)	1.3 (0.5,3.5)
	1990	12	0.06	0.06 (0.03,0.11)	1.2 (0.5,3.3)
	1991	10	0.05	0.05 (0.03,0.10)	1.1 (0.4,3.0)
	1992	6	0.03	0.03 (0.01,0.07)	0.7 (0.2,2.2)
	1993	8	0.04	0.05 (0.02,0.09)	0.9 (0.3,2.7)
	1994	11	0.06	0.06 (0.03,0.11)	1.2 (0.5,3.3)
	1995	7	0.04	0.04 (0.02,0.08)	0.8 (0.2,2.4)
	1996	8	0.04	0.05 (0.02,0.09)	0.9 (0.3,2.7)
	1997	14	0.07	0.08 (0.04,0.13)	1.5 (0.6,4.0)
	1998	19	0.10	0.10 (0.06,0.16)	2.0 (0.9,5.1)
	1999	20	0.10	0.10 (0.06,0.16)	2.1 (0.9,5.2)
	2000	18	0.09	0.09 (0.06,0.15)	1.9 (0.8,4.8)
	2001	13	0.07	0.07 (0.04,0.12)	1.4 (0.5,3.6)
	2002	10	0.05	0.05 (0.03,0.10)	1.1 (0.4,3.0)
	2003	26	0.13	0.14 (0.09,0.20)	2.7 (1.2,6.7)
	2004	21	0.11	0.11 (0.07,0.17)	2.2 (0.9,5.4)
	2005	25	0.12	0.13 (0.08,0.19)	2.6 (1.2,6.4)
	2006	23	0.11	0.11 (0.07,0.17)	2.2 (1.0,5.5)
	2007	20	0.10	0.10 (0.06,0.16)	2.0 (0.9,5.1)
	2008	18	0.09	0.09 (0.05,0.14)	1.7 (0.7,4.4)
	2009	33	0.16	0.16 (0.11,0.23)	3.2 (1.5,7.7)
	2010	20	0.10	0.10 (0.06,0.15)	1.9 (0.8,4.8)
	2011	25	0.12	0.12 (0.08,0.17)	2.3 (1.0,5.7)
	2012	19	0.09	0.09 (0.05,0.14)	1.7 (0.8,4.4)
	2013	27	0.13	0.13 (0.08,0.19)	2.5 (1.2,6.2)
	2014	24	0.11	0.11 (0.07,0.16)	2.2 (1.0,5.4)
	2015	27	0.12	0.12 (0.08,0.18)	2.4 (1.1,5.9)
	2016	24	0.11	0.11 (0.07,0.16)	2.1 (1.0,5.3)

Table S9xiv: Age-standardised incidence rates and rate ratios by calendar period of diagnosis for neuroendocrine carcinomas

Cancer histological type	Calendar year of diagnosis	Number of cases	Crude incidence rate per 100,000 PY	Age-standardised incidence rate per 100,000 PY (95% CI)	Age-standardised incidence rate ratio (95% CI)
Neuroendocrine	1988	29	0.15	0.16 (0.10,0.23)	1.0 [Ref]
	1989	21	0.11	0.11 (0.07,0.17)	0.7 (0.4,1.3)
	1990	13	0.07	0.07 (0.03,0.11)	0.4 (0.2,0.8)
	1991	11	0.06	0.06 (0.03,0.11)	0.4 (0.2,0.8)
	1992	12	0.06	0.07 (0.03,0.12)	0.4 (0.2,0.9)
	1993	13	0.07	0.07 (0.04,0.13)	0.5 (0.2,0.9)
	1994	7	0.04	0.04 (0.02,0.08)	0.2 (0.1,0.6)
	1995	16	0.08	0.09 (0.05,0.15)	0.6 (0.3,1.1)
	1996	13	0.07	0.06 (0.03,0.11)	0.4 (0.2,0.8)
	1997	8	0.04	0.04 (0.02,0.08)	0.3 (0.1,0.6)
	1998	10	0.05	0.05 (0.02,0.09)	0.3 (0.1,0.7)
	1999	17	0.09	0.09 (0.05,0.14)	0.6 (0.3,1.1)
	2000	9	0.05	0.05 (0.02,0.09)	0.3 (0.1,0.7)
	2001	11	0.06	0.05 (0.03,0.10)	0.3 (0.2,0.7)
	2002	14	0.07	0.07 (0.04,0.12)	0.4 (0.2,0.9)
	2003	8	0.04	0.04 (0.02,0.08)	0.3 (0.1,0.6)
	2004	5	0.03	0.03 (0.01,0.06)	0.2 (0.1,0.4)
	2005	18	0.09	0.09 (0.05,0.14)	0.6 (0.3,1.1)
	2006	13	0.06	0.07 (0.03,0.11)	0.4 (0.2,0.8)
	2007	20	0.10	0.10 (0.06,0.16)	0.6 (0.3,1.2)
	2008	19	0.09	0.09 (0.05,0.14)	0.6 (0.3,1.1)
	2009	12	0.06	0.06 (0.03,0.10)	0.4 (0.2,0.8)
	2010	15	0.07	0.07 (0.04,0.12)	0.5 (0.2,0.9)
	2011	19	0.09	0.09 (0.05,0.14)	0.6 (0.3,1.1)
	2012	33	0.16	0.15 (0.10,0.21)	1.0 (0.6,1.7)
	2013	23	0.11	0.11 (0.07,0.16)	0.7 (0.4,1.2)
	2014	25	0.12	0.12 (0.07,0.17)	0.7 (0.4,1.3)
	2015	23	0.11	0.10 (0.07,0.16)	0.7 (0.4,1.2)
	2016	26	0.12	0.11 (0.07,0.17)	0.7 (0.4,1.3)

Table S9xv: Age-standardised incidence rates and rate ratios by calendar period of diagnosis for inflammatory carcinomas

Cancer histological type	Calendar year of diagnosis	Number of cases	Crude incidence rate per 100,000 PY	Age-standardised incidence rate per 100,000 PY (95% CI)	Age-standardised incidence rate ratio (95% CI)
Inflammatory	1988	3	0.02	0.02 (0.004,0.05)	1.0 [Ref]
	1989	5	0.03	0.03 (0.01,0.06)	1.5 (0.3,9.7)
	1990	4	0.02	0.02 (0.01,0.06)	1.3 (0.2,8.7)
	1991	7	0.04	0.04 (0.02,0.08)	2.2 (0.5,13.1)
	1992	6	0.03	0.03 (0.01,0.07)	1.8 (0.4,11.6)
	1993	11	0.06	0.07 (0.03,0.12)	3.7 (1.0,20.6)
	1994	16	0.08	0.08 (0.05,0.13)	4.5 (1.3,24.7)
	1995	28	0.15	0.14 (0.09,0.20)	7.7 (2.4,40.5)
	1996	16	0.08	0.09 (0.05,0.14)	4.8 (1.4,26.2)
	1997	14	0.07	0.08 (0.04,0.13)	4.2 (1.2,23.1)
	1998	23	0.12	0.12 (0.08,0.19)	6.8 (2.1,35.9)
	1999	24	0.12	0.13 (0.08,0.20)	7.3 (2.2,38.2)
	2000	21	0.11	0.11 (0.07,0.17)	5.9 (1.8,31.6)
	2001	13	0.07	0.07 (0.04,0.12)	3.8 (1.1,21.3)
	2002	23	0.12	0.12 (0.08,0.18)	6.6 (2.0,34.6)
	2003	21	0.11	0.10 (0.06,0.16)	5.6 (1.7,30.0)
	2004	9	0.05	0.05 (0.02,0.09)	2.6 (0.7,15.3)
	2005	12	0.06	0.06 (0.03,0.10)	3.3 (0.9,18.4)
	2006	6	0.03	0.03 (0.01,0.06)	1.6 (0.3,10.1)
	2007	15	0.07	0.07 (0.04,0.12)	4.0 (1.2,22.1)
	2008	6	0.03	0.03 (0.01,0.06)	1.6 (0.4,10.2)
	2009	9	0.04	0.04 (0.02,0.08)	2.3 (0.6,13.6)
	2010	14	0.07	0.07 (0.04,0.11)	3.7 (1.1,20.5)
	2011	5	0.02	0.02 (0.01,0.06)	1.3 (0.3,8.6)
	2012	9	0.04	0.04 (0.02,0.08)	2.3 (0.6,13.5)
	2013	7	0.03	0.03 (0.01,0.07)	1.8 (0.4,11.2)
	2014	15	0.07	0.07 (0.04,0.12)	3.8 (1.1,21.0)
	2015	8	0.04	0.03 (0.02,0.07)	1.9 (0.5,11.6)
	2016	4	0.02	0.02 (0.01,0.05)	1.0 (0.2,7.2)

Table S10: Characteristics of 246,477 women diagnosed with invasive breast cancer in England during 2010-2016, grouped according to most common cancer histological types

Characteristic*	Histological type, %							Total %	Total number of women
	Ductal	Lobular	Mucinous	Tubular	Medullary	Rare subtypes†	Unspecified		
**Age at diagnosis** (*p* < 0.001)									
18-39	5.5	1.5	2.2	1.4	16.4	4.1	5.0	4.9	12,081
40-49	18.1	13.5	9.9	16.9	24.6	11.8	14.7	17.2	42,516
50-64	36.9	35.5	21.7	51.7	33.3	30.6	25.3	36.3	89,456
65-70	15.5	18.7	16.7	19.4	11.5	17.7	11.3	15.9	39,228
71-79	13.8	17.5	23.6	7.7	8.7	20.5	17.3	14.5	35,686
80-89	10.2	13.3	25.9	2.9	5.5	15.3	26.4	11.2	27,510
**Cancer screen detected** (*p* < 0.001)									
Eligible: screen detected	27.3	26.5	21.3	55.6	18.5	20.5	9.4	26.9	66,423
Eligible: not screen detected (interval cancer)	11.5	14.1	7.6	5.4	14.5	13.6	10.1	11.6	28,640
Eligible: not screen detected (other)	13.7	13.6	9.5	10.1	11.7	14.2	17.0	13.6	33,621
Not eligible for screening	47.5	45.8	61.6	28.9	55.3	51.7	63.5	47.9	117,793
**Breast cancer laterality** (*p* < 0.001)									
Left	51.1	52.3	52.2	51.0	54.6	53.2	44.3	51.1	125,985
Right	48.6	47.1	47.6	48.8	45.2	46.4	40.5	48.1	118,656
Unknown	0.3	0.6	0.2	0.2	0.2	0.4	15.2	0.8	1836
**Index of multiple deprivation** (*p* < 0.001)									
<20%, least deprived	22.9	24.7	20.4	21.6	20.1	22.9	21.7	23.0	56,736
20-39%	22.7	23.7	22.5	24.0	22.2	21.3	21.9	22.8	56,118
40-59%	20.9	21.3	21.4	22.0	22.7	20.9	21.1	21.0	51,753
60-79%	18.3	17.5	18.8	17.9	18.3	19.2	19.8	18.3	44,999
80+%, most deprived	15.2	12.8	16.9	14.5	16.7	15.7	15.5	14.9	36,871
**Region** (*p* < 0.001)									
Eastern	11.7	12.2	12.2	10.1	11.9	13.4	7.2	11.6	28,653
North West	13.1	12.0	13.6	10.3	8.9	10.0	10.1	12.8	31,495
Northern & Yorkshire	12.7	12.4	12.9	16.4	13.6	15.9	9.0	12.7	31,277
Oxford	5.7	6.1	5.7	6.5	5.9	6.4	6.7	5.8	14,310
South West	15.2	16.4	15.0	17.7	15.9	13.3	11.4	15.2	37,552
Thames	21.6	20.3	20.5	12.8	13.1	19.1	29.1	21.4	52,843
Trent	9.5	9.4	10.3	14.0	14.3	12.1	16.2	9.8	24,210
West Midlands	10.5	11.2	9.8	12.2	16.4	9.8	10.3	10.7	26,137
**Ethnicity** (*p* < 0.001)									
White	81.2	83.8	78.7	84.2	82.0	83.6	71.2	81.3	200,349
South Asian	2.9	1.8	3.8	1.8	4.9	2.9	2.0	2.8	6878
Black	1.9	1.0	2.6	0.7	4.0	1.8	1.9	1.8	4395
Chinese	0.3	0.2	0.4	0.1	0.2	0.3	0.3	0.3	707
Other	1.6	1.2	1.3	1.3	0.7	1.4	1.8	1.6	3908
Unknown	12.1	12.0	13.2	11.9	8.2	10.0	22.8	12.2	30,240
**Tumour grade** (*p* < 0.001)									
Low	14.5	4.9	32.2	95.0	0.0	13.9	6.5	14.8	36,491
Medium	46.7	83.6	59.2	2.9	4.9	38.8	23.7	49.7	122,510
High	36.5	8.1	4.3	0.2	93.2	36.4	23.0	31.7	78,237
Unknown	2.3	3.4	4.3	1.9	1.9	10.9	46.8	3.8	9239
**Stage** (*p* < 0.001)									
Local	37.7	28.4	43.8	78.0	34.2	35.0	14.8	36.7	90,552
Regional	41.5	48.6	36.7	9.6	50.1	46.2	23.5	41.3	101,842
Metastatic	3.5	4.8	1.8	0.6	0.9	2.8	13.3	3.8	9368
Unknown	17.3	18.2	17.7	11.8	14.8	16.0	48.4	18.2	44,715
**Tumour size** (*p* < 0.001)									
1-20 mm	46.6	33.1	45.5	84.4	43.8	41.1	17.6	44.8	110,451
21-50 mm	30.2	34.7	31.5	5.8	45.7	34.8	14.9	30.1	74,193
>50 mm	3.4	11.4	3.3	0.2	2.1	7.5	2.4	4.3	10,590
Unknown	19.8	20.8	19.7	9.6	8.4	16.6	65.1	20.8	51,243
**Number of positive nodes** (*p* < 0.001)									
0	46.0	44.9	58.9	76.5	61.4	54.0	14.8	46.0	113,323
1 to 3	19.8	17.5	7.1	7.1	19.2	14.0	9.2	18.7	46,028
4 to 9	4.8	4.9	1.3	0.6	3.3	3.8	2.7	4.6	11,305
10 or more	2.2	4.3	0.4	0.1	1.4	1.9	1.2	2.3	5691
Unknown	27.2	28.4	32.3	15.7	14.7	26.3	72.1	28.4	70,130
**ER status** (*p* < 0.001)									
Negative	12.2	2.2	1.6	0.3	45.0	30.0	8.4	10.9	26,754
Positive	61.0	73.1	71.0	78.1	25.1	44.8	30.8	61.8	152,342
Unknown	26.8	24.7	27.4	21.6	29.9	25.2	60.8	27.3	67,381
**PR status** (*p* < 0.001)									
Negative	13.1	7.1	4.3	4.0	43.1	24.1	9.1	12.2	30,140
Positive	25.3	28.4	28.9	28.1	4.7	17.6	12.9	25.3	62,365
Unknown	61.6	64.5	66.8	67.9	52.2	58.3	78.0	62.5	153,972
**HER2 status** (*p* < 0.001)									
Negative	62.5	72.1	68.5	79.2	66.3	65.7	31.8	63.3	155,930
Positive	13.6	4.6	4.5	1.0	10.5	8.9	10.1	12.0	29,663
Unknown	23.9	23.3	27.0	19.8	23.2	25.4	58.1	24.7	60,884
**Total %**	**100**	**100**	**100**	**100**	**100**	**100**	**100**	**100**	**-**
**Total number of women**	**198,455**	**28,401**	**4960**	**3993**	**427**	**3923**	**6318**	**-**	**246,477**

* *p* values are for χ^2 tests of independence between known characteristic values and common histological types

† All subtypes that are not mentioned in the table (including "Other"). See Table S11 for details.

Table S11: Characteristics of 3923 women diagnosed with rare histological types of invasive breast cancer in England during 2010-2016

Characteristic*	Rare histological type, %									Total % of rare subtypes
	Papillary	Metaplastic	Cribriform	Apocrine	Micro-papillary	Adenoid cystic	Neuro-endocrine	Inflamm-atory	Other	
**Age at diagnosis** ($p < 0.001$)										
18-39	2.1	5.8	4.4	2.1	4.1	8.4	1.2	12.9	7.1	4.1
40-49	7.4	12.2	21.4	9.1	13.0	16.3	11.0	21.0	15.0	11.8
50-64	27.7	29.8	32.8	30.9	35.5	34.3	34.1	24.2	20.7	30.6
65-70	19.9	14.5	15.9	20.8	18.8	15.1	17.1	21.0	17.1	17.7
71-79	26.0	19.8	15.5	19.3	18.5	13.9	18.9	14.5	24.3	20.5
80-89	16.9	17.9	10.0	17.8	10.1	12.0	17.7	6.4	15.8	15.3
**Cancer screen detected** ($p < 0.001$)										
Eligible: screen detected	20.7	10.1	30.6	25.7	31.5	21.1	18.3	1.6	14.3	20.5
Eligible: not screen detected (interval cancer)	12.3	18.5	9.6	13.2	9.8	12.7	15.2	19.4	10.0	13.6
Eligible: not screen detected (other)	14.6	15.6	8.5	12.8	13.0	15.7	17.7	24.2	13.6	14.2
Not eligible for screening	52.4	55.8	51.3	48.4	45.7	50.6	48.8	54.9	62.1	51.7
**Breast cancer laterality** ($p = 0.89$)										
Left	52.7	53.5	52.0	53.7	55.5	50.0	51.2	46.8	52.1	53.2
Right	46.8	46.1	48.0	46.3	44.1	49.4	47.0	53.2	47.1	46.4
Unknown	0.5	0.4	0.0	0.0	0.4	0.6	1.8	0.0	0.8	0.4
**Index of multiple deprivation** ($p = 0.34$)										
<20%, least deprived	24.6	22.0	24.4	23.5	22.6	23.5	18.9	19.4	20.7	22.9
20-39%	22.0	18.9	23.2	22.4	21.0	21.7	23.2	16.1	27.1	21.3
40-59%	21.2	21.6	21.0	16.3	20.9	19.3	24.4	29.0	25.0	20.9
60-79%	18.7	20.3	19.6	19.1	19.3	17.5	18.9	17.7	15.7	19.2
80+%, most deprived	13.5	17.2	11.8	18.7	16.2	18.0	14.6	17.8	11.5	15.7
**Region** ($p < 0.001$)										
Eastern	16.3	11.6	10.7	11.1	13.6	8.4	23.2	17.7	10.7	13.4
North West	7.8	13.7	10.0	11.1	6.7	13.3	9.1	6.5	9.3	10.0
Northern & Yorkshire	13.1	17.1	10.0	16.7	20.3	14.5	17.1	17.7	13.6	15.9
Oxford	7.4	4.9	5.9	8.0	7.2	6.0	4.9	3.2	5.7	6.4
South West	16.9	14.3	13.3	10.9	7.7	16.9	7.9	8.1	20.7	13.3
Thames	19.2	15.2	21.0	21.6	19.3	18.1	23.2	37.1	22.9	19.1
Trent	10.8	13.5	13.3	12.1	14.2	12.7	5.5	4.8	7.9	12.1
West Midlands	8.5	9.7	15.8	8.5	11.0	10.1	9.1	4.9	9.2	9.8
**Ethnicity** ($p = 0.84$)										
White	80.0	86.6	78.2	84.0	87.2	84.9	83.5	75.8	80.0	83.6
South Asian	3.6	2.0	3.0	3.9	2.8	1.2	3.0	6.5	3.6	2.9
Black	1.9	1.7	2.2	1.6	2.2	1.8	0.6	3.2	0.7	1.8
Chinese	0.3	0.3	0.4	0.4	0.1	0.0	0.6	0.0	0.7	0.3
Other	1.4	1.8	1.8	1.0	1.2	1.2	0.6	0.0	2.1	1.4
Unknown	12.8	7.6	14.4	9.1	6.5	10.9	11.7	14.5	12.9	10.0
**Tumour grade** ($p < 0.001$)										
Low	18.7	4.0	71.6	3.9	4.4	36.7	6.7	1.6	11.4	13.9
Medium	47.5	13.6	24.0	58.4	58.5	28.3	31.7	40.3	51.4	38.8
High	17.9	67.2	1.5	36.0	35.0	13.3	45.7	33.9	25.7	36.4
Unknown	15.9	15.2	2.9	1.7	2.1	21.7	15.9	24.2	11.5	10.9
**Stage** ($p < 0.001$)										
Local	42.5	18.5	62.4	39.9	41.9	33.1	18.3	3.2	37.1	35.0
Regional	34.0	63.7	24.7	42.6	47.3	50.0	40.9	53.2	46.4	46.2
Metastatic	0.5	3.7	1.1	3.1	2.2	0.6	8.5	21.0	2.9	2.8
Unknown	23.0	14.1	11.8	14.4	8.6	16.3	32.3	22.6	13.6	16.0
**Tumour size** ($p < 0.001$)										
1-20 mm	49.7	19.6	63.1	47.9	54.3	35.5	33.5	3.2	42.1	41.1
21-50 mm	29.4	50.0	18.1	30.9	28.5	43.4	33.5	22.6	34.3	34.8
>50 mm	4.7	14.3	1.5	4.7	6.0	7.8	7.9	4.8	6.4	7.5
Unknown	16.2	16.1	17.3	16.5	11.2	13.3	25.1	69.4	17.2	16.6
**Number of positive nodes** ($p < 0.001$)										
0	52.4	61.3	64.9	51.4	44.5	75.3	40.2	6.5	58.6	54.0
1 to 3	12.6	11.5	8.9	17.9	23.2	1.2	14.0	9.7	11.4	14.0
4 to 9	2.7	1.7	1.8	5.3	9.5	0.6	3.0	3.2	2.1	3.8
10 or more	0.8	1.1	0.0	4.1	4.7	0.6	1.2	1.6	1.4	1.9
Unknown	31.5	24.4	24.4	21.3	18.1	22.3	41.6	79.0	26.5	26.3
**ER status** ($p < 0.001$)										
Negative	5.3	58.7	2.2	58.8	6.1	63.3	25.0	25.8	22.1	30.0
Positive	65.3	14.7	71.6	20.2	75.6	7.8	52.4	50.0	45.7	44.8
Unknown	29.4	26.6	26.2	21.0	18.3	28.9	22.6	24.2	32.2	25.2
**PR status** ($p < 0.001$)										
Negative	4.8	46.6	3.0	45.7	8.9	41.0	18.9	17.7	15.7	24.1
Positive	24.9	6.0	28.4	8.2	29.9	4.8	18.9	21.0	18.6	17.6
Unknown	70.3	47.4	68.6	46.1	61.2	54.2	62.2	61.3	65.7	58.3
**HER2 status** ($p < 0.001$)										
Negative	57.9	71.9	71.2	61.5	68.3	70.5	65.9	45.2	65.0	65.7
Positive	6.1	3.6	1.8	19.1	19.7	0.0	3.7	27.4	0.7	8.9
Unknown	36.0	24.5	27.0	19.4	12.0	29.5	30.4	27.4	34.3	25.4
**Total %**	**100**	**100**	**100**	**100**	**100**	**100**	**100**	**100**	**100**	**100**
**Total number of women**	**924**	**1025**	**271**	**486**	**685**	**166**	**164**	**62**	**140**	**3923**

* *p* values are for χ^2 tests of independence between characteristic and rare histological types

Table S12: Distribution of molecular subtype in 246,477 women diagnosed with invasive breast cancer in England during 2010-2016, according to cancer histological types (see Figure 5)

Cancer histological type*	Distribution of molecular subtype, n (%)						Total %	Total number of women
	Luminal A	Luminal B (HER2-)	Luminal B (HER2+)	Basal-like	HER2+ (non-luminal)	Unknown		
Tubular	2805 (70.2)	7 (0.2)	34 (0.9)	2 (0.1)	0 (0.0)	1145 (28.6)	100	3993
Cribriform	167 (61.6)	2 (0.7)	4 (1.5)	2 (0.7)	1 (0.4)	95 (35.1)	100	271
Lobular	16,483 (58.0)	1232 (4.3)	981 (3.5)	237 (0.8)	90 (0.3)	9378 (33.1)	100	28,401
Mucinous	2801 (56.5)	93 (1.9)	154 (3.1)	15 (0.3)	19 (0.4)	1878 (37.8)	100	4960
Micropapillary	274 (40.0)	113 (16.5)	91 (13.3)	13 (1.9)	16 (2.3)	178 (26.0)	100	685
Papillary	346 (37.4)	66 (7.1)	43 (4.7)	16 (1.7)	5 (0.5)	448 (48.6)	100	924
Ductal	71,179 (35.9)	23,695 (11.9)	15,818 (8.0)	10,241 (5.2)	4484 (2.3)	73,038 (36.7)	100	198,455
Other	43 (30.7)	7 (5.0)	0 (0.0)	16 (11.4)	1 (0.7)	73 (52.2)	100	140
Neuroendocrine	45 (27.4)	20 (12.2)	4 (2.4)	21 (12.8)	1 (0.6)	73 (44.6)	100	164
Inflammatory	9 (14.5)	6 (9.7)	9 (14.5)	3 (4.8)	4 (6.5)	31 (50.0)	100	62
Unspecified	836 (13.2)	305 (4.8)	280 (4.4)	211 (3.3)	94 (1.5)	4592 (72.8)	100	6318
Apocrine	45 (9.3)	23 (4.7)	25 (5.1)	138 (28.4)	35 (7.2)	220 (45.3)	100	486
Adenoid Cystic	12 (7.2)	0 (0.0)	0 (0.0)	58 (34.9)	0 (0.0)	96 (57.9)	100	166
Metaplastic	30 (2.9)	110 (10.7)	8 (0.8)	357 (34.8)	12 (1.2)	508 (49.6)	100	1025
Medullary	5 (1.2)	84 (19.7)	17 (4.0)	118 (27.6)	7 (1.6)	196 (45.9)	100	427
**Total %**	**38.6**	**10.5**	**7.1**	**4.6**	**1.9**	**37.3**	**100**	**-**
**Total number of women**	**95,080**	**25,763**	**17,468**	**11,448**	**4769**	**91,949**	**-**	**246,477**

*Cancer histological types are ordered by the percentage of Luminal A cancers

Table S13: Distribution of molecular subtype in 246,477 women diagnosed with invasive breast cancer in England during 2010-2016, according to cancer histological types, separately for age groups at diagnosis (see Figure S3)

Age group & cancer histological type*	Distribution of molecular subtype, n (%)						Total %	Total number of women
	Luminal A	Luminal B (HER2-)	Luminal B (HER2+)	Basal-like	HER2+ (non-luminal)	Unknown		
**<50 years old at diagnosis**								
Tubular	506 (69.4)	2 (0.3)	5 (0.7)	0 (0.0)	0 (0.0)	216 (29.6)	100	729
Cribriform	43 (61.4)	0 (0.0)	2 (2.9)	0 (0.0)	0 (0.0)	25 (35.7)	100	70
Lobular	2464 (57.6)	230 (5.4)	166 (3.9)	21 (0.5)	17 (0.4)	1382 (32.2)	100	4280
Mucinous	310 (51.7)	25 (4.2)	39 (6.5)	0 (0.0)	2 (0.3)	224 (37.3)	100	600
Micropapillary	33 (28.2)	26 (22.2)	26 (22.2)	3 (2.6)	3 (2.6)	26 (22.2)	100	117
Ductal	13,229 (28.2)	6763 (14.4)	5092 (10.8)	3017 (6.4)	1268 (2.7)	17,574 (37.5)	100	46,943
Other	8 (25.8)	0 (0.0)	0 (0.0)	6 (19.4)	0 (0.0)	17 (54.8)	100	31
Papillary	21 (24.1)	13 (14.9)	7 (8.0)	3 (3.4)	1 (1.1)	42 (48.5)	100	87
Apocrine	7 (13.0)	3 (5.6)	4 (7.4)	13 (24.1)	8 (14.8)	19 (35.1)	100	54
Unspecified	123 (9.9)	79 (6.3)	76 (6.1)	62 (5.0)	33 (2.7)	872 (70.0)	100	1245
Inflammatory	2 (9.5)	2 (9.5)	4 (19.0)	1 (4.8)	1 (4.8)	11 (52.4)	100	21
Adenoid Cystic	3 (7.3)	0 (0.0)	0 (0.0)	15 (36.6)	0 (0.0)	23 (56.1)	100	41
Metaplastic	5 (2.7)	25 (13.6)	5 (2.7)	60 (32.6)	1 (0.5)	88 (47.9)	100	184
Medullary	1 (0.6)	38 (21.7)	4 (2.3)	50 (28.6)	1 (0.6)	81 (46.2)	100	175
Neuroendocrine	0 (0.0)	4 (20.0)	0 (0.0)	6 (30.0)	0 (0.0)	10 (50.0)	100	20
**All types**	**16,755 (30.7)**	**7210 (13.2)**	**5430 (9.9)**	**3257 (6.0)**	**1335 (2.4)**	**20,610 (37.8)**	**100**	**54,597**
**50-70 years old at diagnosis**								
Tubular	2020 (71.1)	3 (0.1)	28 (1.0)	1 (<0.1)	0 (0.0)	789 (27.8)	100	2841
Cribriform	90 (68.2)	0 (0.0)	2 (1.5)	1 (0.8)	0 (0.0)	39 (29.5)	100	132
Lobular	9578 (62.2)	604 (3.9)	535 (3.5)	121 (0.8)	47 (0.3)	4515 (29.3)	100	15,400
Mucinous	1159 (60.8)	37 (1.9)	88 (4.6)	8 (0.4)	11 (0.6)	602 (31.7)	100	1905
Micropapillary	158 (42.5)	60 (16.1)	49 (13.2)	4 (1.1)	9 (2.4)	92 (24.7)	100	372
Papillary	177 (40.2)	30 (6.8)	25 (5.7)	10 (2.3)	3 (0.7)	195 (44.3)	100	440
Ductal	41,591 (39.9)	11,704 (11.2)	8087 (7.8)	4965 (4.8)	2349 (2.3)	35,445 (34.0)	100	104,141
Neuroendocrine	30 (35.7)	10 (11.9)	2 (2.4)	5 (6.0)	1 (1.2)	36 (42.8)	100	84
Other	17 (32.1)	4 (7.5)	0 (0.0)	6 (11.3)	1 (1.9)	25 (47.2)	100	53
Unspecified	374 (16.2)	133 (5.8)	132 (5.7)	93 (4.0)	45 (1.9)	1533 (66.4)	100	2310
Inflammatory	3 (10.7)	3 (10.7)	4 (14.3)	1 (3.6)	3 (10.7)	14 (50.0)	100	28
Adenoid Cystic	7 (8.5)	0 (0.0)	0 (0.0)	26 (31.7)	0 (0.0)	49 (59.8)	100	82
Apocrine	21 (8.4)	13 (5.2)	17 (6.8)	66 (26.3)	18 (7.2)	116 (46.1)	100	251
Metaplastic	11 (2.4)	52 (11.5)	2 (0.4)	159 (35.0)	7 (1.5)	223 (49.2)	100	454
Medullary	2 (1.0)	37 (19.4)	13 (6.8)	55 (28.8)	5 (2.6)	79 (41.4)	100	191
**All types**	**55,238 (42.9)**	**12,690 (9.9)**	**8984 (7.0)**	**5521 (4.3)**	**2499 (1.9)**	**43,752 (34.0)**	**100**	**128,684**
**71+ years old at diagnosis**								
Tubular	279 (66.0)	2 (0.5)	1 (0.2)	1 (0.2)	0 (0.0)	140 (33.1)	100	423
Mucinous	1332 (54.3)	31 (1.3)	27 (1.1)	7 (0.3)	6 (0.2)	1052 (42.8)	100	2455
Lobular	4441 (50.9)	398 (4.6)	280 (3.2)	95 (1.1)	26 (0.3)	3481 (39.9)	100	8721
Cribriform	34 (49.3)	2 (2.9)	0 (0.0)	1 (1.4)	1 (1.4)	31 (45.0)	100	69
Micropapillary	83 (42.3)	27 (13.8)	16 (8.2)	6 (3.1)	4 (2.0)	60 (30.6)	100	196
Papillary	148 (37.3)	23 (5.8)	11 (2.8)	3 (0.8)	1 (0.3)	211 (53.0)	100	397
Ductal	16,359 (34.5)	5228 (11.0)	2639 (5.6)	2259 (4.8)	867 (1.8)	20,019 (42.3)	100	47,371
Other	18 (32.1)	3 (5.4)	0 (0.0)	4 (7.1)	0 (0.0)	31 (55.4)	100	56
Inflammatory	4 (30.8)	1 (7.7)	1 (7.7)	1 (7.7)	0 (0.0)	6 (46.1)	100	13
Neuroendocrine	15 (25.0)	6 (10.0)	2 (3.3)	10 (16.7)	0 (0.0)	27 (45.0)	100	60
Unspecified	339 (12.3)	93 (3.4)	72 (2.6)	56 (2.0)	16 (0.6)	2187 (79.1)	100	2763
Apocrine	17 (9.4)	7 (3.9)	4 (2.2)	59 (32.6)	9 (5.0)	85 (46.9)	100	181
Adenoid Cystic	2 (4.7)	0 (0.0)	0 (0.0)	17 (39.5)	0 (0.0)	24 (55.8)	100	43
Metaplastic	14 (3.6)	33 (8.5)	1 (0.3)	138 (35.7)	4 (1.0)	197 (50.9)	100	387
Medullary	2 (3.3)	9 (14.8)	0 (0.0)	13 (21.3)	1 (1.6)	36 (59.0)	100	61
**All types**	**23,087 (36.5)**	**5863 (9.3)**	**3054 (4.8)**	**2670 (4.2)**	**935 (1.5)**	**27,587 (43.7)**	**100**	**63,196**

* Cancer histological types are ordered by the percentage of Luminal A cancers in each age group

Table S14: Distribution of molecular subtype in 246,477 women diagnosed with invasive breast cancer in England during 2010-2016, according to cancer histological types, separately for screen-detection status (see Figure S4)

Screen-detection status & cancer histological type*	Distribution of molecular subtype, n (%)						Total %	Total number of women
	Luminal A	Luminal B (HER2-)	Luminal B (HER2+)	Basal-like	HER2+ (non-luminal)	Unknown		
**Eligible: screen detected**								
Tubular	1587 (71.4)	2 (0.1)	21 (0.9)	1 (<0.1)	0 (0.0)	611 (27.6)	100	2222
Cribriform	56 (67.5)	0 (0.0)	2 (2.4)	0 (0.0)	0 (0.0)	25 (30.1)	100	83
Lobular	4976 (66.0)	205 (2.7)	227 (3.0)	37 (0.5)	14 (0.2)	2080 (27.6)	100	7539
Mucinous	687 (65.1)	15 (1.4)	44 (4.2)	2 (0.2)	4 (0.4)	304 (28.7)	100	1056
Micropapillary	107 (49.5)	30 (13.9)	28 (13.0)	0 (0.0)	3 (1.4)	48 (22.2)	100	216
Ductal	26,118 (48.3)	4705 (8.7)	3549 (6.6)	1625 (3.0)	732 (1.4)	17,397 (32.0)	100	54,126
Papillary	91 (47.6)	11 (5.8)	9 (4.7)	3 (1.6)	1 (0.5)	76 (39.8)	100	191
Neuroendocrine	13 (43.3)	6 (20.0)	0 (0.0)	0 (0.0)	0 (0.0)	11 (36.7)	100	30
Other	8 (40.0)	2 (10.0)	0 (0.0)	1 (5.0)	0 (0.0)	9 (45.0)	100	20
Unspecified	193 (32.4)	31 (5.2)	39 (6.5)	20 (3.4)	4 (0.7)	309 (51.8)	100	596
Adenoid Cystic	5 (14.3)	0 (0.0)	0 (0.0)	9 (25.7)	0 (0.0)	21 (60.0)	100	35
Apocrine	9 (7.2)	3 (2.4)	5 (4.0)	37 (29.6)	7 (5.6)	64 (51.2)	100	125
Metaplastic	5 (4.8)	12 (11.5)	0 (0.0)	41 (39.4)	2 (1.9)	44 (42.4)	100	104
Medullary	1 (1.3)	17 (21.5)	7 (8.9)	23 (29.1)	1 (1.3)	30 (37.9)	100	79
Inflammatory	0 (0.0)	0 (0.0)	0 (0.0)	0 (0.0)	0 (0.0)	1 (100.0)	100	1
**All types**	**33,856 (51.0)**	**5039 (7.6)**	**3931 (5.9)**	**1799 (2.7)**	**768 (1.2)**	**21,030 (31.6)**	**100**	**66,423**
**Eligible: not screen detected (interval cancer)**								
Cribriform	19 (73.1)	0 (0.0)	0 (0.0)	0 (0.0)	0 (0.0)	7 (26.9)	100	26
Tubular	153 (70.5)	1 (0.5)	1 (0.5)	0 (0.0)	0 (0.0)	62 (28.5)	100	217
Lobular	2434 (60.7)	219 (5.5)	149 (3.7)	46 (1.1)	17 (0.4)	1142 (28.6)	100	4007
Mucinous	227 (59.9)	13 (3.4)	19 (5.0)	3 (0.8)	1 (0.3)	116 (30.6)	100	379
Micropapillary	29 (43.3)	12 (17.9)	7 (10.4)	2 (3.0)	2 (3.0)	15 (22.4)	100	67
Neuroendocrine	9 (36.0)	3 (12.0)	1 (4.0)	3 (12.0)	0 (0.0)	9 (36.0)	100	25
Papillary	35 (30.7)	14 (12.3)	11 (9.6)	5 (4.4)	2 (1.8)	47 (41.2)	100	114
Ductal	6776 (29.7)	3457 (15.2)	2102 (9.2)	1805 (7.9)	818 (3.6)	7845 (34.4)	100	22,803
Other	4 (28.6)	0 (0.0)	0 (0.0)	3 (21.4)	1 (7.1)	6 (42.9)	100	14
Inflammatory	2 (16.7)	1 (8.3)	2 (16.7)	1 (8.3)	0 (0.0)	6 (50.0)	100	12
Unspecified	81 (12.7)	56 (8.8)	46 (7.2)	41 (6.4)	23 (3.6)	392 (61.3)	100	639
Apocrine	5 (7.8)	6 (9.4)	10 (15.6)	15 (23.4)	4 (6.3)	24 (37.5)	100	64
Adenoid Cystic	1 (4.8)	0 (0.0)	0 (0.0)	8 (38.1)	0 (0.0)	12 (57.1)	100	21
Metaplastic	2 (1.1)	21 (11.1)	0 (0.0)	67 (35.3)	2 (1.1)	98 (51.4)	100	190
Medullary	0 (0.0)	13 (21.0)	4 (6.5)	19 (30.6)	1 (1.6)	25 (40.3)	100	62
**All types**	**9777 (34.1)**	**3816 (13.3)**	**2352 (8.2)**	**2018 (7.0)**	**871 (3.0)**	**9806 (34.4)**	**100**	**28,640**
**Eligible: not screen detected (other)**								
Tubular	280 (69.7)	0 (0.0)	6 (1.5)	0 (0.0)	0 (0.0)	116 (28.8)	100	402
Cribriform	15 (65.2)	0 (0.0)	0 (0.0)	1 (4.3)	0 (0.0)	7 (30.5)	100	23
Lobular	2168 (56.3)	180 (4.7)	159 (4.1)	38 (1.0)	16 (0.4)	1293 (33.5)	100	3854
Mucinous	245 (52.1)	9 (1.9)	25 (5.3)	3 (0.6)	6 (1.3)	182 (38.8)	100	470
Papillary	51 (37.8)	5 (3.7)	5 (3.7)	2 (1.5)	0 (0.0)	72 (53.3)	100	135
Ductal	8697 (32.0)	3542 (13.0)	2436 (9.0)	1535 (5.6)	799 (2.9)	10,203 (37.5)	100	27,212
Neuroendocrine	8 (27.6)	1 (3.4)	1 (3.4)	2 (6.9)	1 (3.4)	16 (55.3)	100	29
Other	5 (26.3)	2 (10.5)	0 (0.0)	2 (10.5)	0 (0.0)	10 (52.7)	100	19
Micropapillary	22 (24.7)	18 (20.2)	14 (15.7)	2 (2.2)	4 (4.5)	29 (32.7)	100	89
Apocrine	7 (11.3)	4 (6.5)	2 (3.2)	14 (22.6)	7 (11.3)	28 (45.1)	100	62
Unspecified	100 (9.3)	46 (4.3)	47 (4.4)	32 (3.0)	18 (1.7)	832 (77.3)	100	1075
Inflammatory	1 (6.7)	2 (13.3)	2 (13.3)	0 (0.0)	3 (20.0)	7 (46.7)	100	15
Adenoid Cystic	1 (3.8)	0 (0.0)	0 (0.0)	9 (34.6)	0 (0.0)	16 (61.6)	100	26
Metaplastic	4 (2.5)	19 (11.9)	2 (1.3)	51 (31.9)	3 (1.9)	81 (50.5)	100	160
Medullary	1 (2.0)	7 (14.0)	2 (4.0)	13 (26.0)	3 (6.0)	24 (48.0)	100	50
**All types**	**11,605 (34.5)**	**3835 (11.4)**	**2701 (8.0)**	**1704 (5.1)**	**860 (2.6)**	**12,916 (38.4)**	**100**	**33,621**

(table continued)

(table continued)

Screen-detection status & cancer histological type*	Distribution of molecular subtype, n (%)						Total %	Total number of women
	Luminal A	Luminal B (HER2-)	Luminal B (HER2+)	Basal-like	HER2+ (non-luminal)	Unknown		
**Not eligible for screening**								
Tubular	785 (68.1)	4 (0.3)	6 (0.5)	1 (0.1)	0 (0.0)	356 (31.0)	100	1152
Cribriform	77 (55.4)	2 (1.4)	2 (1.4)	1 (0.7)	1 (0.7)	56 (40.4)	100	139
Mucinous	1642 (53.7)	56 (1.8)	66 (2.2)	7 (0.2)	8 (0.3)	1276 (41.8)	100	3055
Lobular	6905 (53.1)	628 (4.8)	446 (3.4)	116 (0.9)	43 (0.3)	4863 (37.5)	100	13,001
Micropapillary	116 (37.1)	53 (16.9)	42 (13.4)	9 (2.9)	7 (2.2)	86 (27.5)	100	313
Papillary	169 (34.9)	36 (7.4)	18 (3.7)	6 (1.2)	2 (0.4)	253 (52.4)	100	484
Ductal	29,588 (31.4)	11,991 (12.7)	7731 (8.2)	5276 (5.6)	2135 (2.3)	37,593 (39.8)	100	94,314
Other	26 (29.9)	3 (3.4)	0 (0.0)	10 (11.5)	0 (0.0)	48 (55.2)	100	87
Neuroendocrine	15 (18.8)	10 (12.5)	2 (2.5)	16 (20.0)	0 (0.0)	37 (46.2)	100	80
Inflammatory	6 (17.6)	3 (8.8)	5 (14.7)	2 (5.9)	1 (2.9)	17 (50.1)	100	34
Unspecified	462 (11.5)	172 (4.3)	148 (3.7)	118 (2.9)	49 (1.2)	3059 (76.4)	100	4008
Apocrine	24 (10.2)	10 (4.3)	8 (3.4)	72 (30.6)	17 (7.2)	104 (44.3)	100	235
Adenoid Cystic	5 (6.0)	0 (0.0)	0 (0.0)	32 (38.1)	0 (0.0)	47 (55.9)	100	84
Metaplastic	19 (3.3)	58 (10.2)	6 (1.1)	198 (34.7)	5 (0.9)	285 (49.8)	100	571
Medullary	3 (1.3)	47 (19.9)	4 (1.7)	63 (26.7)	2 (0.8)	117 (49.6)	100	236
**All types**	**39,842 (33.8)**	**13,073 (11.1)**	**8484 (7.2)**	**5927 (5.0)**	**2270 (1.9)**	**48,197 (41.0)**	**100**	**117,793**

* Cancer histological types are ordered by the percentage of Luminal A cancers in each screen-detection status group

Table S15: Characteristics of 198,455 women diagnosed with invasive ductal carcinoma in England during 2010-2016, grouped according to molecular subtypes

Characteristic*	Molecular subtype, %						Total %	Total number of women
	Luminal A	Luminal B (HER2-)	Luminal B (HER2+)	Basal-like	HER2+ (non-luminal)	Unknown		
**Age at diagnosis (*p* < 0.001)**								
18-39	18.4	15.8	12.7	9.2	3.4	40.5	100	10,935
40-49	31.2	14.0	10.3	5.6	2.5	36.4	100	36,008
50-64	38.7	11.4	8.3	4.8	2.4	34.4	100	73,296
65-70	42.9	10.9	6.5	4.7	1.9	33.1	100	30,845
71-79	36.4	12.0	6.2	5.1	2.1	38.2	100	27,292
80-89	32.0	9.7	4.8	4.3	1.5	47.7	100	20,079
**Cancer screen detected (*p* < 0.001)**								
Eligible: screen detected	48.3	8.7	6.6	3.0	1.4	32.0	100	54,126
Eligible: not screen detected (interval cancer)	29.7	15.2	9.2	7.9	3.6	34.4	100	22,803
Eligible: not screen detected (other)	32.0	13.0	9.0	5.6	2.9	37.5	100	27,212
Not eligible for screening	31.4	12.7	8.2	5.6	2.3	39.8	100	94,314
**Breast cancer laterality (*p* < 0.001)**								
Left	35.6	11.9	8.0	5.3	2.3	36.9	100	101,380
Right	36.4	12.0	8.0	5.0	2.2	36.4	100	96,406
Unknown	5.7	2.5	2.7	1.2	0.6	87.3	100	669
**Index of multiple deprivation (*p* < 0.001)**								
<20%, least deprived	36.2	11.6	7.9	4.6	2.2	37.5	100	45,497
20-39%	36.8	12.4	8.2	4.9	2.1	35.6	100	45,011
40-59%	36.2	12.0	7.9	5.0	2.2	36.7	100	41,517
60-79%	35.2	12.0	8.0	5.3	2.4	37.1	100	36,317
80+%, most deprived	34.3	11.5	7.9	6.3	2.6	37.4	100	30,113
**Region (*p* < 0.001)**								
Eastern	44.5	18.1	10.2	6.6	2.7	17.9	100	23,153
North West	34.7	10.5	7.4	6.2	2.8	38.4	100	25,924
Northern & Yorkshire	46.2	13.4	9.9	6.0	2.4	22.1	100	25,214
Oxford	34.2	12.3	7.8	3.2	1.5	41.0	100	11,326
South West	39.1	12.9	9.0	3.5	1.6	33.9	100	30,141
Thames	22.6	7.7	5.1	4.0	1.8	58.8	100	42,918
Trent	32.7	10.1	6.8	4.7	1.8	43.9	100	18,903
West Midlands	41.6	14.0	9.3	7.6	3.7	23.8	100	20,876
**Ethnicity (*p* < 0.001)**								
White	38.2	12.6	8.5	5.4	2.3	33.0	100	161,158
South Asian	28.2	12.6	8.2	6.5	3.2	41.3	100	5836
Black	22.6	13.7	7.8	8.4	3.2	44.3	100	3737
Chinese	32.2	10.3	12.3	5.5	3.8	35.9	100	603
Other	28.8	11.6	9.6	6.3	3.1	40.6	100	3271
Unknown	25.1	7.1	4.3	2.5	1.2	59.8	100	23,850
**Tumour grade (*p* < 0.001)**								
Low grade	64.9	0.0	1.9	0.4	0.1	32.7	100	28,767
Medium grade	56.6	0.0	6.7	1.8	1.1	33.8	100	92,682
High grade	0.0	32.7	12.1	11.5	4.5	39.2	100	72,445
Unknown	0.0	0.0	6.1	2.7	2.2	89.0	100	4561
**Stage (*p* < 0.001)**								
Local	48.7	8.8	6.4	4.3	1.5	30.3	100	74,757
Regional	31.7	16.5	9.9	7.2	3.1	31.6	100	82,312
Metastatic	23.4	12.0	11.0	5.3	4.1	44.2	100	6935
Unknown	20.4	8.0	6.2	2.3	1.5	61.6	100	34,451
**Tumour size (*p* < 0.001)**								
1-20 mm	46.2	9.4	6.9	4.0	1.6	31.9	100	92,528
21-50 mm	29.2	17.4	9.5	6.8	2.6	34.5	100	60,031
>50 mm	23.4	17.1	10.1	7.9	4.4	37.1	100	6724
Unknown	23.9	8.5	7.8	4.9	2.9	52.0	100	39,172
**Number of positive nodes (*p* < 0.001)**								
0	45.2	11.9	7.5	5.5	1.7	28.2	100	91,270
1 to 3	36.3	14.9	8.8	4.4	2.4	33.2	100	39,212
4 to 9	27.2	18.2	10.5	5.6	3.5	35.0	100	9487
10 or more	20.5	19.2	11.8	6.4	5.1	37.0	100	4283
Unknown	22.5	8.2	7.4	4.9	2.6	54.4	100	54,203
**Total %**	**35.9**	**11.9**	**8.0**	**5.2**	**2.3**	**36.7**	**100**	**-**
**Total number of women**	**71,179**	**23,695**	**15,818**	**10,241**	**4484**	**73,038**	**-**	**198,455**

* *p* values are for χ^2 tests of independence between known characteristic and molecular subtypes

Results: Supplementary Figures

Figure S3: Percentage of molecular subtype within each histological type for 246,477 women diagnosed during 2010-2016, split by age group at diagnosis. The vertical lines show percentages for all types combined**.** Histological types are ordered by the percentage of Luminal A cancers (see **Table S13** for numbers).

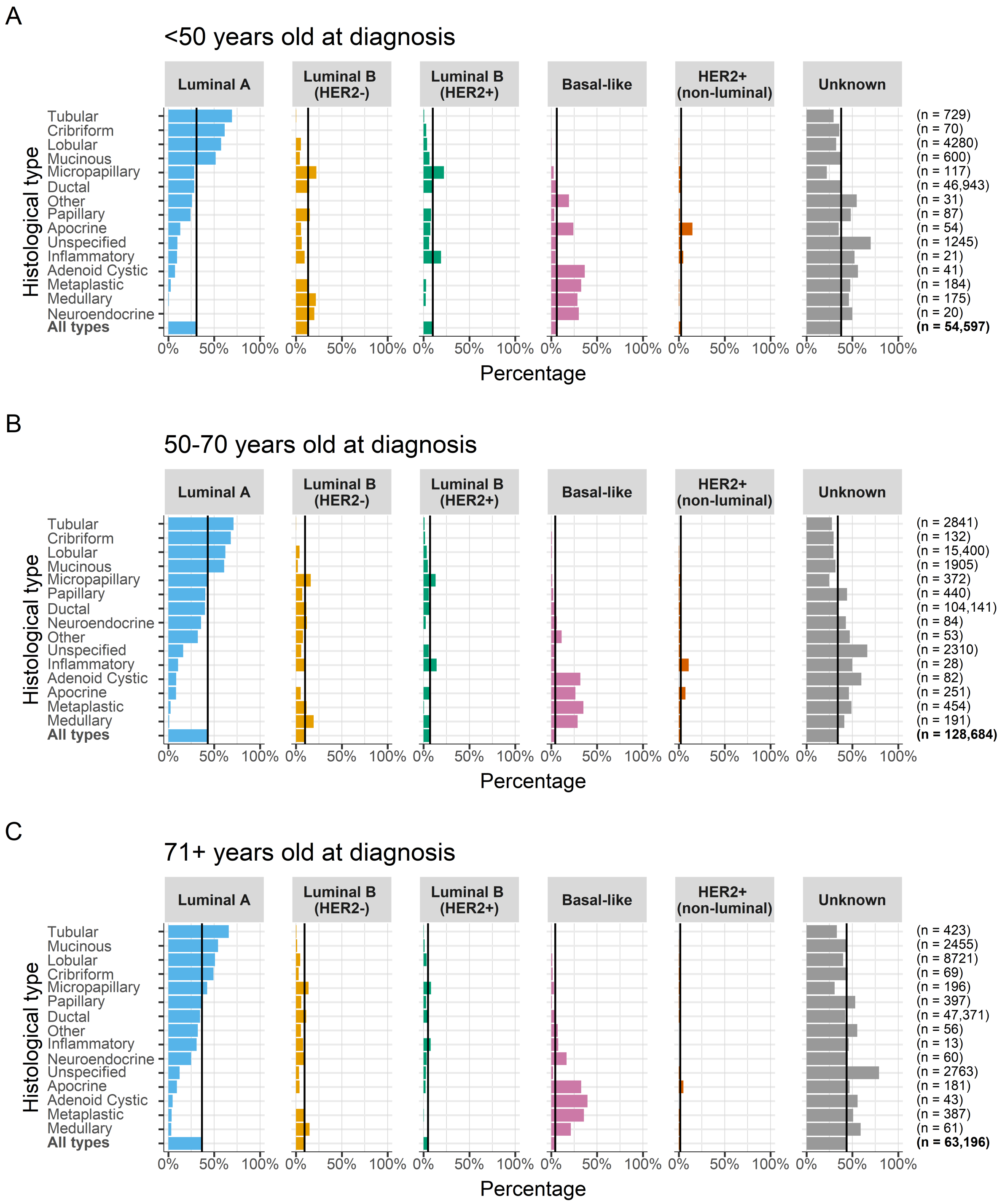

Figure S4: Percentage of molecular subtype within each histological type for 246,477 women diagnosed during 2010-2016, split by screen-detection status. The vertical lines show percentages for all types combined**.** Histological types are ordered by the percentage of Luminal A cancers within each screen-detection subgroup. Categories of screen-detection reflect eligibility for breast cancer screening programme (i.e. 50-64 years for all calendar periods of diagnosis and 65-70 years from 2005) (see **Table S14** for numbers).

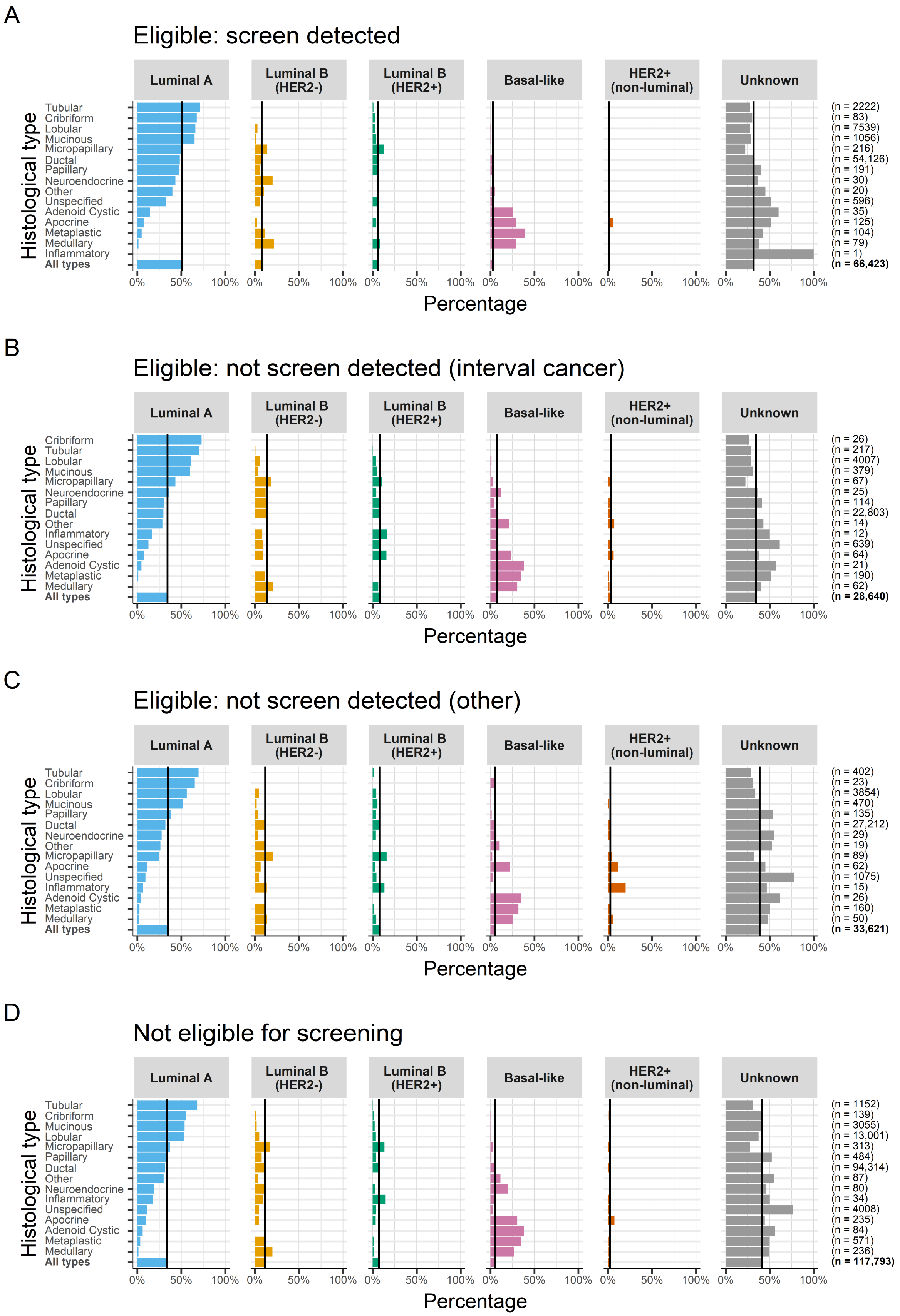

Supplement: Supplementary file 1 — Figure S1. Composition of the study population among women diagnosed with invasive breast cancer in England during January 1988 to December 2016 Figure S2. Histological types of invasive breast carcinoma Text S1. Explanatory note regarding breast cancer histopathology classification Table S1. Summary of the changes in breast cancer classification over time, according to WHO Classification Editions Table S2. ICD‐O3 codes for histological types of invasive breast cancer Table S3. Approximations for breast cancer molecular subtypes using immunohistochemistry Table S4. Age‐specific incidence rates (per 100,000 person‐years) and corresponding incidence rate ratios of 838,776 women diagnosed with invasive breast cancer in England during 1988–2016, according to each cancer histological type Table S5. Age distribution (%) of 838,776 women diagnosed with invasive breast cancer in England during 1988–2016, according to cancer histological types Table S6. Characteristics of 838,776 women diagnosed with invasive breast cancer in England during 1988–2016, grouped according to most common cancer histological types Table S7. Characteristics of 10,616 woman diagnosed with rare histological types of breast cancer during 1988–2016 Table S8. Distribution of screen‐detection status in 838,776 women diagnosed with invasive breast cancer in England during 1988–2016, according to cancer histological types Table S9. Age‐standardised incidence rates and rate ratios by calendar period of diagnosis by cancer histological type Table S10. Characteristics of 246,477 women diagnosed with invasive breast cancer in England during 2010–2016, grouped according to most common cancer histological types Table S11. Characteristics of 3923 women diagnosed with rare histological types of invasive breast cancer in England during 2010–2016 Table S12. Distribution of molecular subtype in 246,477 women diagnosed with invasive breast cancer in England during 2010–2016, according to cancer histological types Tab [file CJP2-11-e70043-s001.pdf]
